# The interplay of fibroblasts, the extracellular matrix, and inflammation in scar formation

**DOI:** 10.1016/j.jbc.2021.101530

**Published:** 2021-12-23

**Authors:** Leandro Moretti, Jack Stalfort, Thomas Harrison Barker, Daniel Abebayehu

**Affiliations:** Department of Biomedical Engineering, University of Virginia, Charlottesville, Virginia, USA

**Keywords:** fibroblast, fibronectin, fibrin, extracellular matrix, transforming growth factor beta, integrin, fetal and adult wound healing, macrophage, mast cell, interleukin, αSMA, alpha-smooth muscle actin, 9III, the ninth type III repeat in Fn, 10III, the 10th type III repeat in Fn, BMP, bone morphogenic protein, cFn, cell-secreted Fn, DAMP, damage-associated molecular pattern, DC, dendritic cell, ECM, extracellular matrix, EDA, extra domain type III A, EDB, extra domain type III B, FAK, focal adhesion kinase, FBG, fibrinogen, Fn, fibronectin, GPI, glycosylphosphatidylinositol, HA, hyaluronic acid, HGF, hepatocyte growth factor, HMGB1, high mobility group box 1, IBD, inflammatory bowel disease, IL, interleukin, IPF, idiopathic pulmonary fibrosis, LEMD3, LEM domain–containing protein 3, MMP, metalloproteinase, NET, neutrophil extracellular trap, PAD, protein arginine deiminase, PDGF, platelet-derived growth factor, pFn, plasma Fn, SFK, Src family kinase, SMAD, small mothers against decapentaplegic, SPARC, secreted protein acidic and rich in cysteine, TGF-β, transforming growth factor beta, Thy-1, thymocyte differentiation antigen 1, TNC, tenascin C, TNF, tumor necrosis factor, TREM1, triggering receptors expressed on myeloid cells-1, TSP, thrombospondin, VEGF, vascular endothelial growth factor, Vn, vitronectin, Wnt, wingless type

## Abstract

Various forms of fibrosis, comprising tissue thickening and scarring, are involved in 40% of deaths across the world. Since the discovery of scarless functional healing in fetuses prior to a certain stage of development, scientists have attempted to replicate scarless wound healing in adults with little success. While the extracellular matrix (ECM), fibroblasts, and inflammatory mediators have been historically investigated as separate branches of biology, it has become increasingly necessary to consider them as parts of a complex and tightly regulated system that becomes dysregulated in fibrosis. With this new paradigm, revisiting fetal scarless wound healing provides a unique opportunity to better understand how this highly regulated system operates mechanistically. In the following review, we navigate the four stages of wound healing (hemostasis, inflammation, repair, and remodeling) against the backdrop of adult *versus* fetal wound healing, while also exploring the relationships between the ECM, effector cells, and signaling molecules. We conclude by singling out recent findings that offer promising leads to alter the dynamics between the ECM, fibroblasts, and inflammation to promote scarless healing. One factor that promises to be significant is fibroblast heterogeneity and how certain fibroblast subpopulations might be predisposed to scarless healing. Altogether, reconsidering fetal wound healing by examining the interplay of the various factors contributing to fibrosis provides new research directions that will hopefully help us better understand and address fibroproliferative diseases, such as idiopathic pulmonary fibrosis, liver cirrhosis, systemic sclerosis, progressive kidney disease, and cardiovascular fibrosis.

Properly resolving wound healing in adults is a complex and tightly regulated process, both spatially and temporally. Sometimes, adult wound healing degenerates toward fibrosis, a process characterized by chronic inflammation, aberrant extracellular matrix (ECM) deposition, and myofibroblast accumulation. The latter develop functional characteristics typical of contractile cells and appear in the context of wound closure and contraction (1). This fibroproliferative response leads to the formation of scar tissue that has increased stiffness and decreased extensibility, and that recovers little, if any, of the original function (2, 3, 4). While adult wound healing typically ends with scar formation, characterized as *ex novo* connective tissue with myofibroblasts and collagen fibers (5), it has long been demonstrated that fetal would healing ends in scarless regeneration. The fetal dermis during early gestation can regenerate wounds without a scar because of the ECM being deposited in such a way that resembles the original tissue and restores functional features, such as sebaceous glands and follicles (6). In prenatal humans, this capability lasts until week 24 (7) and until day 18.5 in rats (8). While this difference in healing outcomes has been known for quite some time, recent discoveries and advances warrant revisiting adult and fetal wound healing conditions. Better understanding of the complex interplay between the ECM, fibroblasts, and inflammation might be leveraged to promote tissue regeneration and avoid, or perhaps even reverse, fibrosis. The wound healing process generally involves four different phases occupying various timescales after injury occurs. In chronological order, these stages are hemostasis, inflammation, repair, and remodeling (Fig. 1).

**Figure 1:**
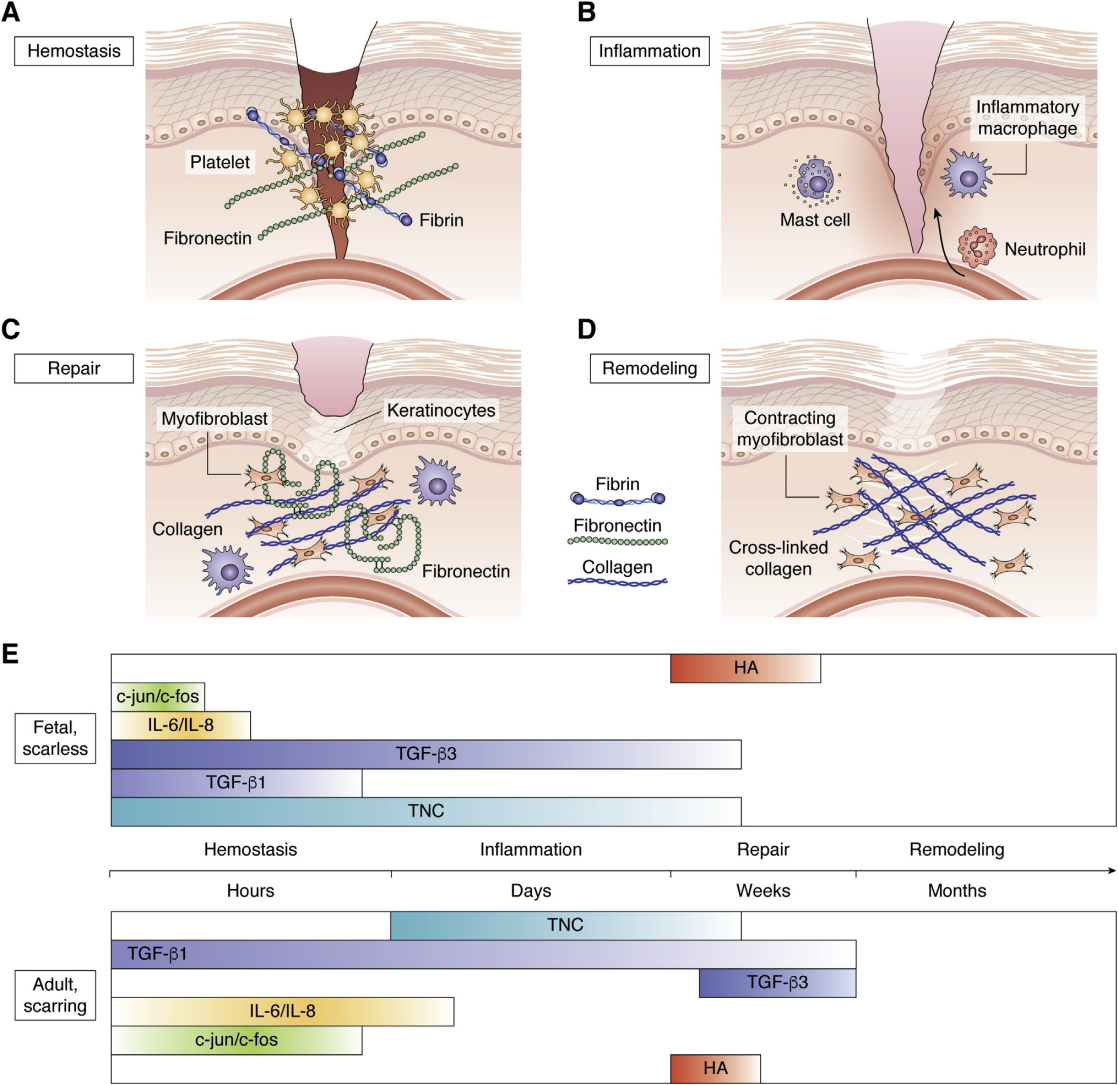
**Phases of wound healing and temporal expression of healing factors in fetal scarless and adult wounds.***A*, hemostasis takes place right after injury. Platelets stop blood loss by activating, releasing their granules, and forming an early provisional matrix made up of fibrin and fibronectin. *B*, approximately 2 h after injury, inflammatory signals released by platelets and tissue-resident immune cells, such as macrophages, and mast cells recruit neutrophils from circulation to the site of injury. Macrophages at this stage are activated in their proinflammatory state, involving secretion of interleukins IL-6 and IL-8. *C*, at the beginning of repair, the early provisional matrix being used to produce a more mature late provisional matrix made up of collagen and fibronectin. Keratinocytes undergo a proliferative burst to re-epithelialize the wound. Dermal fibroblasts migrate into the wound. First, they secrete fibronectin, then enter an activated state named myofibroblasts, characterized by the expression of alpha-smooth muscle actin and secrete collagen as well. Afterward, keratinocytes migrate over the newly produced ECM and release vascular endothelial growth factor. The ensuing angiogenesis and neovascularization, needed to support myofibroblastic presence, leads to the formation of granulation tissue, characterized by high density of myofibroblasts, macrophages, capillaries, and loosely organized collagen bundles. *D*, remodeling canonically begins once myofibroblasts begin contraction. Myofibroblasts increase expression of actin stress fibers and integrins, in order to produce the contraction needed to realign the excessively deposited ECM, mostly collagen at this stage. Activated fibroblasts continue to support the mechanical load until the ECM is crosslinked, creating striated scar tissue, which in skin becomes paler and paler as time goes by and vascularization is lost. *A*–*D*, adapted from “Wound Healing,” by BioRender.com (2021). Retrieved from https://app.biorender.com/biorender-templates. *E*, the duration of expression or upregulation of select genes and transcription factors in fetal wound healing are represented in the *top half*, whereas the *bottom* represents the duration of the same molecules in adult scarring healing. Hyaluronan (HA) is highly expressed in the wound site for 3 weeks and 1 week in fetal and adult conditions, respectively. c-jun and c-fos, transcription factors for activator protein-1, are quickly upregulated for up to 2 h in fetal wound, whereas in adults, they last past 12 h. Proinflammatory interleukins IL-6 and IL-8 are increased in response to wounds in adults for up to 72 h, but they disappear after just 12 h in fetal scarless wounds. Transforming growth factor beta 1 (TGF-β1, profibrotic) is released and upregulated for 18 h since the wound in the fetus, while its expression is sustained for weeks in the adult. In the case of fibrotic development, TGF-β1 does not subside. Its antifibrotic isoform TGF-β3 is expressed as soon as 10 min after injury in fetal conditions, whereas in adult wounds, it appears after 1 week. Tenascin C (TNC) is also expressed in the fetal wound bed as soon as 1 h after injury, whereas this does not take place until after 24 h in adults.

Hemostasis starts following initial injury, as platelet binding to the newly damaged ECM causes them to release their granules (9). These are made up of several molecules, such as clotting factors, cytokines, and ECM proteins. We will explore the differences between adult and fetal wound healing during the hemostasis phase, with particular focus on inflammatory mediators, fibroblast phenotypes, and ECM components in the early provisional matrix. This matrix is made up by proteins in plasma and released from the platelet granules, with fibrin and fibronectin (Fn) representing the lion's share (10, 11, 12).

The formation of a stable clot and the initiation of leukocyte migration signify the start of the second phase of wound healing, inflammation. Inflammation is initiated by many factors: ATP and nucleic acids released from cells after their injury (13), proinflammatory cytokines from the platelet granules (9, 14), and immune cells recruited by the fibrin matrix (15). Approximately 2 h postinjury, inflammatory signals released by platelets and tissue-resident immune cells, such as macrophages, dendritic cells (DCs), and mast cells, recruit neutrophils to the site of injury (16). Tissue-resident immune cells are innate immune cells that have matured in the peripheral tissue and remain there for the duration of their lifetime. Tissue-resident immune cells secrete various chemokines, which prompt neutrophil recruitment and chemotaxis, that is, migration toward the wound site (Table 1) (17). Typically in wound healing, the recruitment of immune cells and subsequent inflammatory cytokine and chemokine expression is spatially and temporally limited. On the other hand, fibrotic disorders across tissue contexts demonstrate chronic activation of tissue-resident and tissue-recruited immune cells (18, 19). Understanding how the inflammatory phase differs between adult and fetal wound healing will advance two goals: first, identifying inflammatory mediators or cell populations that could be therapeutic targets and second, understanding how inflammation informs fibroblast and ECM biology.

**Table 1:** Relevant cytokines

Cytokine/chemokine	Receptor	Function	Involved in
IL-1⍺/β	IL-1RI & IL-1RAP	Early inflammatory marker and is a pyrogen (292)	Fibrosis (293)
IL-4	IL-4R⍺ & ɣc chain or IL-4R⍺ & IL-13R⍺1	B-cell activation, Th2 differentiation, macrophage polarization (145, 294)	Tissue regeneration, asthma, T-cell immunity (295, 296, 297)
IL-6	IL-6R & gp130	Early inflammatory marker and is a pyrogen (17)	Broad involvement with inflammatory pathologies (17)
IL-8	CXCR1/2	Neutrophil recruitment (298)	Histological inflammation (298)
IL-10	IL-10R⍺/β	Anti-inflammatory processes (116)	Scarless fetal healing (299)
IL-12	IL-12Rβ1/2	Th1 differentiation (300)	T-cell immunity (300)
IL-13	IL-4R⍺ & IL-13R⍺1 or IL-13R⍺2	Th2 differentiation (294, 301)	Tissue regeneration, asthma, T-cell immunity (295, 296, 297, 302)
IL-33	ST2 & IL-1RAP	Alarmin/DAMP (303)	Inflammation, asthma, Macrophage polarization (17, 304)
TNF⍺	TNFR1 or TNFR2	Early inflammatory marker and is a pyrogen (305)	Broad involvement with inflammatory pathologies (17)
IFNɣ	IFNɣR1/2	Macrophage polarization and facilitates antigen presentation (306, 307)	Antigen-specific immunity and viral immunity (307, 308)
MCP-1/CCL2	CCR2	Immune cell recruitment (306)	Tissue repair, infection clearance, and inflammation (306)
MIP-1⍺/CCL3	CCR1/5	Immune cell recruitment (309)	Tissue repair, infection clearance, and inflammation (309)
G-CSF	G-CSFR	Hematopoiesis (17)	Neutrophil maturation and proliferation (17)
CXCL1	CXCR2	Immune cell recruitment (310)	Inflammation and tissue repair (310)
CXCL2	CXCR2	Immune cell recruitment (310)	Inflammation and tissue repair (310)

Abbreviation: MCP-1, monocyte chemoattractant protein-1.

The third phase of wound healing is repair, which is characterized by the early provisional matrix being used to produce a more mature late provisional matrix based on coordination between inflammatory mediators, fibroblasts, and ECM components that now include Fns, proteoglycans, and collagens (12, 20). During primary repair in dermis, the tissue most studied by the field, skin cells known as keratinocytes move from the basal lamina through the provisional matrix. Upon reaching the wound location, keratinocytes undergo a proliferative burst that is stopped only by contact inhibition once the wound is closed. Secondary repair in skin occurs when re-epithelialization is not sufficient (21, 22). In several tissues including dermis, fibroblasts migrate into the wound. First, they secrete Fn, but after transforming growth factor beta (TGF-β, a pleiotropic cytokine with several isoforms) signaling from macrophages (23), they enter an activated state named myofibroblasts, characterized by the expression of alpha-smooth muscle actin (αSMA) (24) and collagen secretion. Myofibroblasts secrete more ECM (mainly collagen and Fn) and contract the wound bed. However, unchecked myofibroblasts can lead to fibrosis. Afterward, keratinocytes migrate over the newly produced ECM and release vascular endothelial growth factor (VEGF) (25). The ensuing angiogenesis and neovascularization, needed to support myofibroblastic presence, leads to the formation of granulation tissue. This tissue derives its name from a high density of myofibroblasts, macrophages, capillaries, and loosely organized collagen bundles (22). While a single marker for pathological myofibroblasts has not been discovered yet, a combination of αSMA, Fn-extra domain type III A (EDA), and lack of thymocyte differentiation antigen 1 (Thy-1, a glycosylphosphatidylinositol [GPI]-anchored protein that modulates certain integrins engagement) could be employed as markers. Such a strategy would enable precise targeting of the effector cells in pathological fibrotic contexts other than skin wounds, where topical therapeutic delivery may be sufficient. We will explore how the repair phase differs between the fibrosis that occurs during adult healing and the scarless fetal wound healing scenario. We will focus on the ECM components, the integrins that they ligate, and fibroblast phenotypes that dictate tissue repair.

With the exception of small dermal wounds, remodeling is the last step of wound healing. This phase, which may last weeks or months, is characterized by the crosslinking of the ECM secreted by myofibroblasts, as well as other modifications that might alter tissue remodeling and function. We will identify the differences during tissue remodeling in adult and fetal wound healing by focusing on differences in cytokine signaling to fibroblasts and enzymatic changes to the ECM.

Given the overall complexity of wound healing, solving fibrotic scarring by recapitulating fetal scarless healing appears to be out of reach with a traditional single target and silver bullet drug. For example, in the indication of idiopathic pulmonary fibrosis (IPF), even the two Food and Drug Administration–approved therapies demonstrate only moderate extension of quality-adjusted life years, with nintedanib and pirfenidone adding 1.41 and 0.73 quality-adjusted life years over standard care, respectively (26). We believe that studies with a more holistic approach with respect to the pathways involved will ensure the field's success in the search for scarless healing. As such, the focus on elucidating the mechanism of fibrosis across organ systems should shift toward the combinatorial effects of the major factors involved: fibroblasts, the ECM, and inflammation, respectively, the *actors*, *stage*, and *setting* of the saga of wound healing. These types of studies could be inspired by the fetal wound healing process, given that a fetus can regenerate wounds without scar tissue into the second trimester of human gestation.

This review compares and contrasts fibrotic wound healing in adults to the ideal standard of fetal scarless healing, attempting to tease out pathways and molecules that, when dysregulated, become biomarkers or promoters of fibrosis. In doing so, we follow the chronological event of the traditional healing response, with emphasis on proteins contributing to hemostasis and the provisional matrix, the mediators of inflammation, and the phenotype of fibroblasts, the effector cells. We conclude by proposing new pathways and areas to investigate in the future.

## Hemostasis and ECM

Hemostasis has two parts: primary hemostasis and secondary hemostasis, both of which aim to stop the loss of blood from the circulation (27). In primary hemostasis, platelets aggregate around the site of injury to form a primary clot. In secondary hemostasis, insoluble fibrin is generated by the coagulation cascade and used to strengthen the primary clot. Understanding the mechanisms and molecules that facilitate these early parts of wound healing is critical to devise therapeutic approaches.

Damage to resident cells and the surrounding ECM caused by the wound initiates the coagulation cascade as well as other pathways that reach well beyond the effort to close the wound. Damaged cells often lyse and release ATP into the extracellular space, initiating inflammation and cell-mediated immunity *via* P2 purinergic signaling (13). At the same time, platelets from circulating plasma bind to the now exposed and damaged endothelial ECM, causing their activation and eventual primary clot formation. Platelets, considered the first responders to sites of vascular injury (9), lack a nucleus; yet possess three organelles: alpha granules, dense granules, and lysosomes. Alpha granules are the key organelles for protein storage, including growth factors, coagulation factors, extracellular adhesion molecules, cytokines, proteoglycans, and chemokines (14). Upon adhesion to the damaged endothelium (collagen fibrils) and their activation, platelets release their characteristic granules. These contain cytokines necessary to mediate inflammation (*e.g.*, tumor necrosis factor [TNF], TNF-α, and interleukin [IL]-1β), the next stages of wound healing, and the coagulation cascade. Key among those released clotting factors are fibrinogen (FBG), prothrombin, factor V, factor XI, factor XIII, von Willebrand factor, and angiopoietin-1, a potent angiogenic cocktail (28). FBG and von Willebrand factor promote platelet activation and aggregation at the injury site, forming a positive feedback loop that increases clotting and helps increase localized tissue factor release.

Tissue factor, expressed by cells that are normally not exposed to flowing blood, contributes to thrombin formation, causing cleavage of FBG into fibrin. Thus, thrombin leads fibrin to polymerize into a fibrin network. Platelets bind to fibrin networks and distinguish between soluble FBG and FBG immobilized on other platelets *via* integrin αIIbβ3 (29, 30). Adhered platelets release biochemical signals that prompt further platelet aggregation and the formation of fibrin from FBG, creating a hemostatic plug (31). Platelets then contract the fibrin network. Clot stiffness is a major factor in bleeding and thrombotic disorders. It was shown that platelet adhesion on stiffer substrates leads to higher levels of platelet activation, including release of alpha granules (32). Typically, platelets match clot and surrounding tissue stiffnesses by synergistically coupling mechanical and biochemical signals. The geometry of the underlying matrix regulates alpha granule secretion and deposition of matrix proteins (33). Alpha granules contain ECM proteins, including thrombospondin (TSP), Fn, vitronectin (Vn), high–molecular weight kininogen, osteonectin (secreted protein acidic and rich in cysteine [SPARC]), plus a variety of protease inhibitors (34). Platelets also release several growth factors: platelet-derived growth factor (PDGF), the proangiogenic VEGF, and TGF-β. The latter is a prominent cytokine with various effects on wound healing and fibrosis development and will be discussed extensively later. In this context however, TGF-β recruits macrophages as well as fibroblasts and stimulates their proliferation, as those cells participate even to the ideal case of scarless wound healing.

Overall, activated and aggregated platelets are critical for providing FBG immobilization and forming the provisional fibrin matrix.

### FBG and fibrin

FBG is a plasma protein that contains two large domains (D regions), connected by fibrillar domains (alpha, beta, and gamma chains) and linear peptide chains. FBG can be polymerized to form fibrin during the formation of the provisional matrix by thrombin-mediated cleavage of fibrin peptide A and fibrin peptide B subunits (Fig. 1*A*). Additional crosslinking methods exist (35).

Both FBG and fibrin have two integrin-binding sites: RGDS and RGDF. While platelets bind those sites with their characteristic integrins αIIb and αIIIa during clot formation, only a small subset of fibroblast integrins can engage those sites. Moreover, studies show that adhesion of fibroblasts on a solely fibrin matrix *in vitro* is reduced (36, 37) compared with the provisional matrix, likely because of the lack of integrin α5β1 engagement. This suggests that in matrices made up of both Fn and fibrin, such as the provisional matrix, fibroblasts will engage preferentially with the Fn integrin–binding domains.

Differences between fetal (up to neonatal) and adult wound healing have been detected in the context of hemostasis, beginning with the *ex vivo* differences between human newborn and adult fibrin clots. In neonates, the “fetal” version of fibrin makes up the FBG network and differs from adult FBG by an increased content of sialic acid (38), which causes neonatal FBG to have a different electrical charge and increased phosphorus content than adult (38, 39). This leads to coagulation of clots that lack three-dimensional structure, are more porous, and are made up of aligned fibers as opposed to the highly crosslinked adult clots (40). Moreover, fetal FBG clots demonstrated an increased rate of degradation, which favors fibrinolysis, and could be implicated in the scarless wound healing typical of fetuses (Table 2).

**Table 2:** Different roles of ECM, cells, and signaling molecules in fetal or adult wound healing

Wound healing component	Contribution to fetal scarless healing	Contribution to scars in adults
ECM		
Collagen	Higher expression levels of collagen III for longer and in a weave pattern (200, 201, 202, 248, 249, 250)	Collagen I is dominant (203)
Fibrin(ogen)	Forms porous clots made by aligned fibers (14, 40, 69)	Clot and early provisional matrix formation (31)
Fn	cFn with EDA, EDB domains is the predominant isoform (57, 58, 62, 311, 312)	Cellular forces can activate the integrin switch toward αvβ3 engagement (50, 53, 54, 55, 56)
Hyaluronan	Expressed in wound for up to 3 weeks (205)	Expression ends within 1 week (205). Can stimulate stress fiber formation (209)
SPARC	Sequesters PDGF isoforms (68)	Indirectly leads to hydrogen peroxide production by fibroblasts (74), contributes to fibroblasts resisting apoptosis (75, 76)
TNC	Expressed earlier and for longer ensuing wound (100, 101)	Implied in organ fibrosis (102); KO attenuates pulmonary and skin fibrosis (107) and suppresses proinflammatory macrophages (108, 109); and activates latent TGF-β1 (97)
Thrombospondin	TSP-1 promotes timely resolution of inflammation (82, 84); TSP-2 delays wound healing but contributes to formation of collagen fibrils (86, 87)	TSP-1 activates TGF-β latency-associated complex (78, 79); TSP-1 is highly expressed in hypertrophic scars (85); and TSP-4 is proinflammatory (88)
Vitronectin	Not essential for development (92, 93)	Clot formation and complement cascade (90, 91)
Cells		
DCs	Subdued inflammation (144)	Necessary for remodeling (143)
Fibroblasts	αSMA is constitutively expressed (185); TGF-β isoforms are expressed at lower levels compared with adult fibroblasts (313); higher levels of p-SMAD intracellularly (166); TGF-β1 autoinduction loop is missing (184); altered prostaglandin E2 pathway (314)	Cofilin-1 and profilin-1 are upregulated compared with fetal fibroblasts (315); PDGFRα+ fibroblasts contribute to fibrotic remodeling (279); PDGFRβ+ fibroblasts' subpopulation emerges in fibrosis (278); express prolyl-4-hydroxylase (204); nitrosylation of caspase3 prevents apoptosis (270); emergence of αSMA+ and HAS1+ subpopulation in pulmonary fibrosis samples (281)
Macrophages	Tissue remodeling activation (17, 126); fetal macrophages resemble anti-inflammatory activation (127); macrophages expressing arginase compete with fibroblasts l-arginine (132, 133, 134, 135)	Proinflammatory activation (17, 126); sustain myofibroblast activation *via* cadherin-11 in fibrosis (138); can transition to myofibroblasts in fibrosis (139, 140, 141)
Mast cells	Less abundant and degranulate less in scarless wounds (117, 118)	Scar formation *via* IL-33 secretion (119) and response to HMGB1 (120)
Neutrophils	Neonatal NIF inhibits NET formation, controlling inflammation (149, 150)	Release superoxide, hydrogen peroxide, inflammatory cytokines, and proteases (147)
Signaling molecules		
BMP	Profibrotic BMP-2 is expressed at lower levels and only in hair follicles (158, 159)	BMP-1 cleaves TSP-1 enhancing TGF-β release promoting myofibroblast activation (157)
HGF	Interferes with TGF-β1 signaling (283)	
TGF-β	Early upregulation of TGF-β3 isoform (168); exogenous TGF-β1 leads to scar even in fetal wounds (166); TGF-β3/TGF-β1 ratio is higher than in adult wounds (177, 178, 179, 180, 181); TGF-β1 is cleared by 18 h (186)	High levels of TGF-β1 and TGF-β2 isoforms (179); temporally limited upregulation of TGF-β3 (after a week) (167); scar stiffness inhibits LEMD3 antagonism of TGF-β (182)
Thy-1	Fetal fibroblasts express Thy-1 (219); regulates engagement of αvβ3 and αvβ5 (212, 213)	Thy-1–negative fibroblasts localized in fibrotic lesions (204, 214)
Wnt	Wnt6 reduces epithelial-to-mesenchymal transition (198)	Responsible for epithelial-to-mesenchymal transition in keloid and dermal scars (189, 190, 191); Wnt3a increases proliferation and collagen I secretion in adult fibroblasts (197)

Fibrin and FBG have also been noted to alter leukocyte recruitment at the site of injury. Fibrin networks are akin to an endogenous hydrogel that results from coagulation and make up a provisional matrix (along with Fn) into which cells migrate and adhere during wound healing (41). Notably, thrombin-catalyzed formation of fibrin results in various peptide fragments, which become soluble ligands available to alter the inflammatory phase of wound healing. For example, fibrin peptide A and fibrin peptide B were shown to increase neutrophil and macrophage recruitment *via* increased expression of monocyte chemoattractant protein-1. The main leukocyte receptor for fibrin is αmβ2/Mac-1; however, fibrin has been reported to be recognized by other receptors depending on the cell recognizing it, such as α5β3, toll-like receptor 4, and vascular endothelial-cadherin (42, 43). Fibrin and FBG have been shown to elicit varying inflammatory responses: increasing macrophage exposure to fibrin promoted increased IL-10 production, whereas treating macrophages with soluble FBG promoted increased TNF production and suppressed IL-10 production (Table 1) (44).

To summarize, the peculiarities of the fetal version of FBG justify targeting FBG in adults in order to recapitulate scarless wound healing. However, systemic KO and inhibition strategies will not succeed, since FBG is fundamental for coagulation.

### Fn

**Figure 2:**
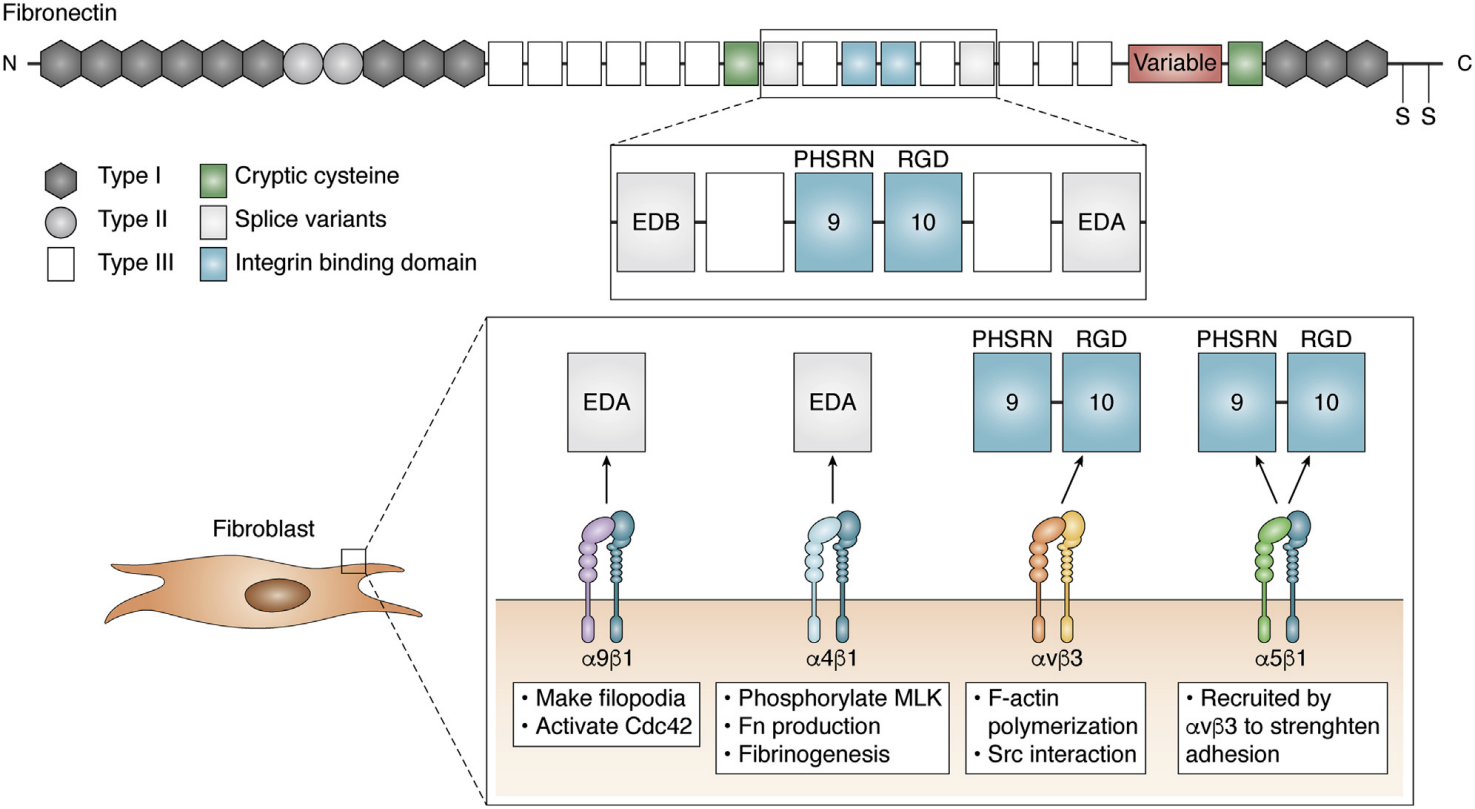
**Fibronectin structure and fibroblast integrins that bind it.***Top*, schematic of a single 220 kDa subunit of fibronectin. Type III repeats making up the integrin-binding domain are in *blue*, extra domain type III repeats that are part of the cellular fibronectin (cFn) isoform are in *gray*. *Green* type III (7th and 15th) domains where glutathionylation may occur under conditions that unfold the protein domains. *Bottom*, the fibroblast integrins binding Fn are the following: α9β1 and α4β1 bind the extra domain A present in cell secreted Fn, whereas αvβ3 and α5β1 bind the canonical integrin-binding domain. While αvβ3 binds the RGD motif on the 10th type III repeat (and other proteins), integrin α5β1 requires the “synergy” PHSRN peptide sequence on the ninth type III to be in close proximity. Cell-generated forces on Fn fibers can unfold the type III repeats, increasing the distance between RGD and the synergy site, inhibiting α5β1 engagement. Under those conditions, only αvβ3 can properly bind the integrin-binding domain of Fn. This phenomenon, first predicted *via* steered molecular dynamics, has been named “integrin switch.”

Fn is a glycoprotein formed by two identical subunits weighing 220 kDa each that are covalently linked by two disulfide bonds toward the C termini. Each subunit is composed of three repeated domain types: type I, type II, and type III (45). Fn interaction with collagen to form the fibrillar components of the ECM (46) is mediated by cell activity, often fibroblasts (47). Among ECM fibular proteins, Fn is of particular importance to the pursuit of scarless healing because of its presence in the provisional matrix of remodeling tissues, its effects on cell behavior through specific integrin interactions, its high degree of spatial flexibility, and its unfolding response to force (Fig. 2).

**Figure 3:**
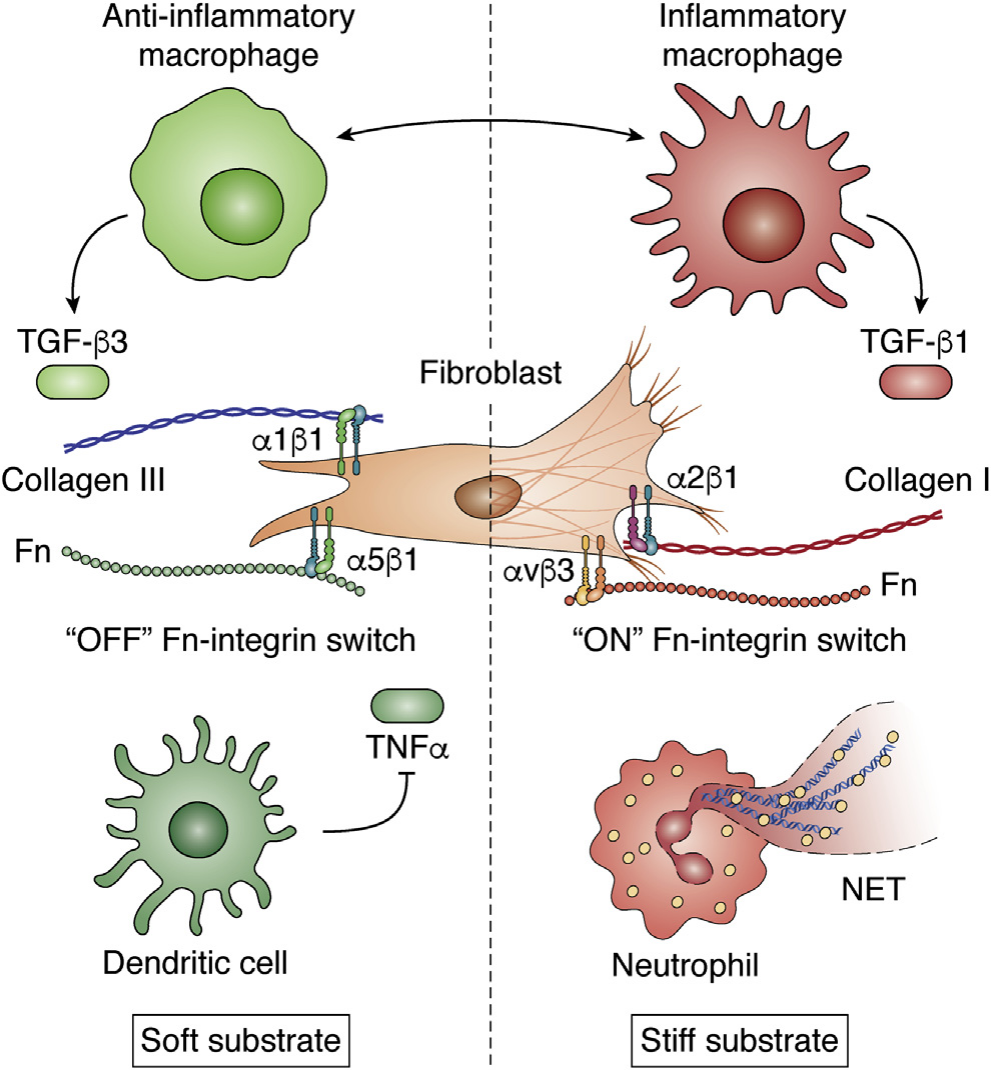
**Main differences between fetal, scarless (*green*, *left side*), and adult scar-producing wound healing.** The fetal ECM is softer than in the adult or postnatal conditions. The predominant macrophage activation state is anti-inflammatory. Macrophages are known to transition between inflammatory, anti-inflammatory, and tissue remodeling. Furthermore, inflammatory macrophages can interact and push fibroblasts to become activate as myofibroblasts (*right*) and secrete the profibrotic isoform TGF-β1. In fetal conditions, the predominant isoform is TGF-β3, which has an antifibrotic effect. Integrin α1β1 binds collagen III, the more abundant collagen isoform in fetal ECM, leading to a downregulation of collagen production. Because of the softer environment, α5β1 can properly bind the integrin-binding domain of fibronectin and maintain fibroblast homeostasis. During adult wound healing, collagen I, a ligand for α2β1, is the most common collagen isoform. Increased cell contractility and ECM mechanics inhibit α5β1 binding of Fn causing an increase in αvβ3 fibroproliferative signaling. Although dendritic cells are present in fetal and adult wound healing, in the fetus, they suppress TNFα secretion, thus subduing inflammation. On the other hand, in the adult and postnatal conditions, neutrophils are recruited to the injury site and are much more likely to release their neutrophil extracellular traps, exacerbating inflammation and leading to downstream scar formation.

Fn type III domains consist of antiparallel β sheets connected by flexible loops and held together by hydrogen bonds (48, 49). Consequently, type III repeats are highly susceptible to force-induced unfolding (50). The 10th type III repeat in Fn (10III) that contains the canonical RGDS sequence was determined to be the type III repeat with the lowest threshold for deformation (51), implying that it will be one of the first domains to unfold under force. This property of 10III can affect the Fn integrin–binding domain, which also comprises the ninth type III repeat (9III). The latter contains a synergistic site with sequence PSHRN that provides domain recognition, mediates cell adhesion, and influences cytoskeletal organization (52, 53). 9III is required by integrin α5β1 to be in close proximity to the RGD motif for proper binding (54). The proximity of these two sites has been the object of several studies because of the importance of physical coupling between extracellular domains and integrin selectivity. Indeed, RGD peptides by themselves are not enough to select specific integrin binding, since they are ubiquitous in other ECM proteins and ligate several kinds of integrin (53). When a force of just 10 pN is applied, the 10III partially unfolds and the RGD domain is removed from the synergy site (50), greatly reducing α5β1 binding (55). Conversely, integrins that do not coordinate binding with the synergy site, such as αvβ3, are not affected by the change in distance (56). The mechanism that can turn on or off some integrin binding through changes in the distance between 9III and 10III has been called the integrin switch (Figs. 2 and 3).

Beyond the 9III–10III integrin switch, Fn contains other variable domains that can affect the wound healing process. The EDA and extra domain type III B (EDB) can be spliced between the 11III and 12III and between the 7III and 8III, respectively. These alternative forms of Fn may include EDA, EDB, or both and are secreted locally by cells, with the exception of hepatocytes that secrete plasma Fn (pFn) in the bloodstream. While levels of cell-secreted Fn (cFn) decrease with age in animals (57, 58), the extra domains are expressed alternatively to contribute to wound healing and related pathologies (Table 2). While knocking out Fn during development is lethal, inducible mouse models allowed the field to tease out the particular effects of pFn and cFn on complex tissue fibrosis diseases. Systemic KO of pFn does not impede clot formation and skin wound healing, most likely because cFn released from platelets at first, and subsequently from fibroblasts and macrophages, maintains Fn levels while platelets decrease in abundance (59, 60). Surprisingly, ablating cFn specifically in mouse fibroblasts is protective against fibrotic scar formation ensuing myocardial infarction and reperfusion. While it must be noted that enough pFn contributed to healing, these data may be explained by the attenuation of pathological cFn and collagen deposition from (cardiac) fibroblasts (61).

Unlike cFn-EDB, cFn-EDA is present in the granules of platelets (62) and contributes to increased thrombosis (63). EDA-containing Fn, produced by fibroblasts and absent in pFn, is needed for the transition from inflammation to the repair phase of wound healing. Consequently, cFn-EDA makes up part of the provisional matrix and favors fibroblast infiltration. Unsurprisingly, it was found that EDA Fn KO mice are protected from bleomycin-induced pulmonary fibrosis, whereas inflammation states and small mothers against decapentaplegic (SMAD) signaling did not differ from wildtype mice. However, injury with a high dose of bleomycin led to higher mortality in EDA KO mice. Given that EDA KO mouse-derived fibroblasts plated on cFn-EDA show the typical response after exposure to TGF-β (64, 65), it can be concluded that this effect is protein specific. This apparent contradiction between low and high bleomycin mouse models is explained by accounting for the unabated inflammation processes. Since EDA is necessary for the transition to the repair phase, knocking it out would continue the secretion of free radicals and localized cell necrosis associated with inflammation. In conclusion, EDA and EDB play essential nuanced roles in development and phases of wound healing, making targeted intervention complicated and prone to side effects.

In addition to the aforementioned findings, EDA is necessary for accumulation of latent TGF-β-binding protein 1 in the ECM, which complexes the inactive form of TGF-β along with the latency-associated peptide (64, 66). The effects of TGF-β signaling span from inflammation to the repair phase of wound healing and are covered further later.

### SPARC, TSP, and Vn

SPARC (a 32 kDa glycoprotein also known as osteonectin), TSP, and Vn are among the ECM proteins released by platelets' alpha granules during clot formation. SPARC is also expressed by fibroblasts downstream of TGF-β signaling (67) and has a multifaceted impact in wound healing. SPARC was reported to sequester two PDGF isoforms, thus inhibiting potent mitogen signaling in fibroblasts (68), whereas depleting SPARC was shown to dysregulate wound healing by altering fibrillar collagen deposition *in vivo* (69, 70, 71, 72). This mechanism was explained by integrin-linked kinase–mediated cell contractility, Fn unfolding, and Fn assembly (73). SPARC-regulated integrin-linked kinase signaling also affects production of hydrogen peroxide by fibroblasts (74), a reactive oxygen species that may contribute to the effects described in the “Oxidative stress” section later. Since redox stress is typically absent in fetal wound healing conditions, SPARC may affect scarring in adults beyond ECM assembly. SPARC not only is detected in scars and fibrotic regions (75, 76) but also contributes apoptosis resistance to fibroblasts contributing to remodeling *via* β-catenin signaling (Table 2) (76).

**Figure 4:**
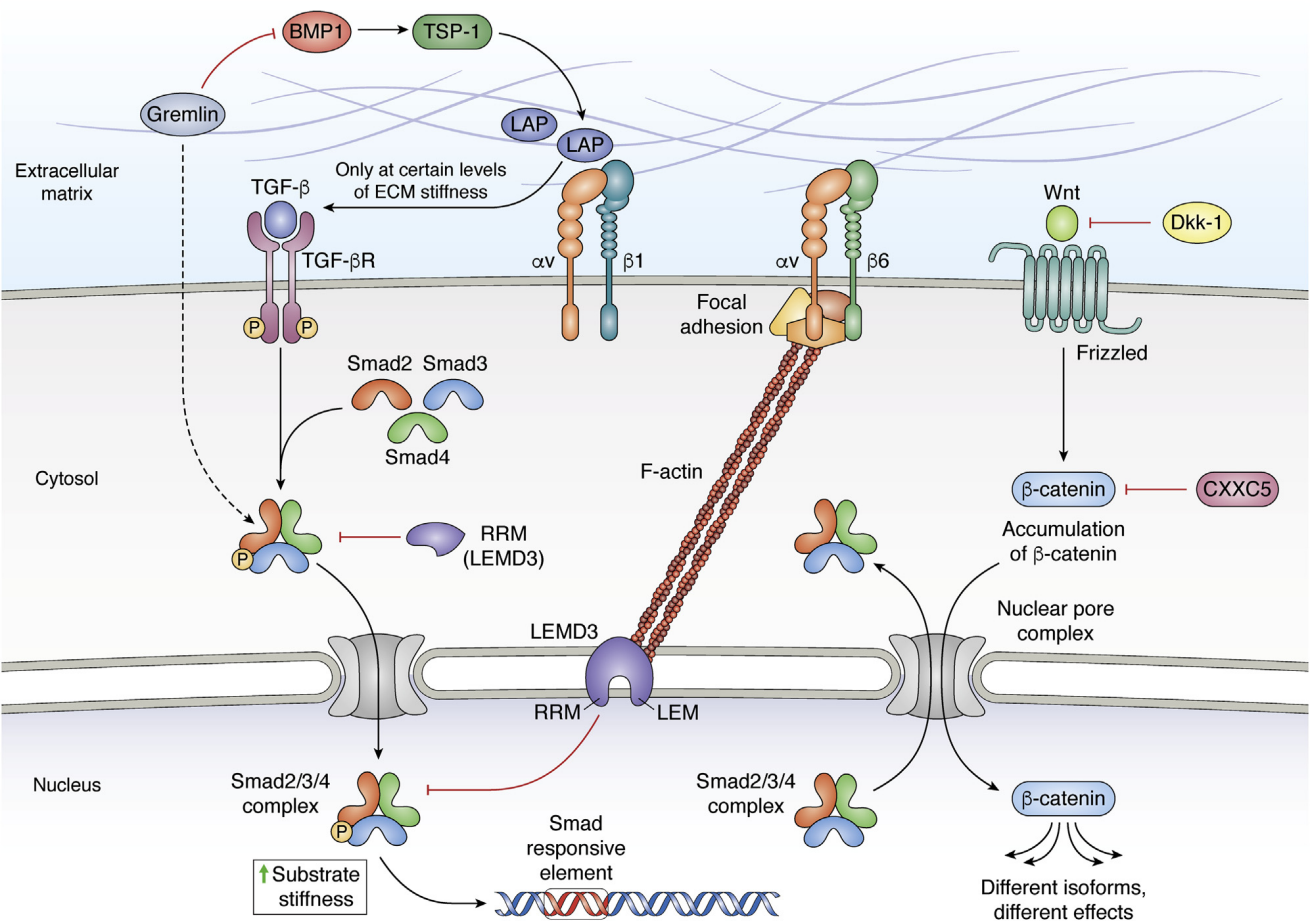
**Summary of TGF-β signaling and interplay with Wnt.** Mechanical cues from the extracellular matrix potentiate TGF-β activation from the latency-associated peptide. In most cases, cells can access TGF-β by releasing it from the complex *via* αv class integrin-mediated forces that are transmitted only at certain levels of ECM stiffness. Alternatively, BMP-1 cleaves TSP-1 and enhances its capability to liberate TGF-β. Gremlin can not only antagonize this BMP-1 activity but also activate SMAD2/3 in the absence of TGF-β. TGF-β ligates its receptors, leading to phosphorylation and formation of Smad2/3/4 complexes that then translocate to the nucleus on chromatin Smad response elements. LEM domain–containing protein 3 (LEMD3) (*purple*) antagonizes this TGF-β/Smad2/3 signaling by complexing with Smad2/3 both in the nuclear envelope and inhibits the mechanical response of cells to TGF-β. Cytosolic LEMD3 fragments are post-translationally generated, separating the nuclear-localizing LEM domain and the Smad2/3-interacting RRM domain (RRM in cytosol, *purple*). Both nuclear and cytosolic LEMD3 activities are inhibited by F-actin polymerization, which is driven by mechanical cues from the matrix, thereby connecting ECM mechanics to inhibition of an inhibitor of Smad2/3 signaling. TGF-β signaling downregulates Dkk-1 expression, which is an antagonist for Wnt. This soluble factor engages its receptor Frizzled, enabling accumulation of β-catenin in the cytosol and thus the latter translocation into the nucleus where it acts as a pluripotent transcription factor. In studies attempting to recapitulate scarless healing, CXXC5 (a zinc finger catenin inhibitor) reduced αSMA and collagen I expression in fibroblasts. Inhibitory relationships are marked with *red block-end arrows*. Wnt, wingless type.

TSPs are five multidomain oligomeric glycoproteins with distinct domain structures and binding sites for cells, growth factors, glycosaminoglycans, and other matrix proteins (77). Analogously to SPARC, TSP-1 is involved in TGF-β signaling by binding to the latency-associated complex and activating it (Fig. 4) (78, 79). Furthermore, SPARC and TSP-1 are expressed by both fibroblasts and macrophages at different stages of the wound healing process, at least in adults (80). TSP-1 increases availability of soluble TGF-β1 *in vitro* and promotes fibroblasts recruitment in *in vivo* wound beds (81). TSP-1 plays a role in many wound healing phases, starting with hemostasis, where it is required for platelet aggregation (82). TSP-1–deficient mice are characterized by expanded and more persistent granulation tissue and increased macrophage presence compared with wildtype littermates. This suggests that TSP-1 is necessary to progress wound healing beyond the inflammatory phase (83). Since TSP-1 KO and TSP-1/TSP-2 double KO mice demonstrated prolonged persistence of inflammation and delayed scab loss (84), TSP-1 is necessary for healing resolution as well. However, human hypertrophic scars are characterized by heightened TSP-1 transcript levels in scar fibroblasts. The same study shows that overexpression of TSP-1 in fibroblasts promotes proliferation, migration, apoptosis resistance, and TGF-β1 expression (85). Taken together, the findings from TSP-1–deficient mice and human hypertrophic scars highlight the importance of tight TSP-1 expression regulation throughout the wound healing process.

TSP-2 KO mice with intact *TSP-1* gene heal and close wounds faster compared with wildtype mice, at the cost of granulation tissue disorganization, characterized by an increased cellular presence (86). This is likely because of TSP-2 contribution to collagen fibril formation (87). Expression of another TSP family member, TSP-4, promotes a proinflammatory phenotype, macrophage accumulation, and adhesion (Table 2) (88).

Vn is a 75 kDa blood plasma protein with binding domains for integrins and matrix proteins. It represents 0.2 to 0.5% of total plasma proteins and is synthesized in the liver. Having an RGD motif, Vn can ligate the integrins made up of the αv subunit and the αIIbβ3 heterodimer of platelets, leading to clot formation (89). Moreover, Vn participates in the complement cascade and regulates proteolytic degradation of the clot *via* binding to plasminogen activation inhibitor-1. These functions are reviewed at length elsewhere (90, 91). Vn has been shown to be not essential for mammalian development, suggesting that the complement cascade and clot formation are solidly redundant processes (92, 93) and that it likely does not contribute to the scarless healing processes typical of fetuses (Table 2).

### Tenascin C

Tenascin C (TNC) is another matricellular protein that mediates cell adhesion and is unique in that it is not constitutively expressed in most adult tissues but is induced at sites of inflammation (94, 95). It is comprised of 200 kDa hexamers and possesses 511 isoforms, since it contains nine splice variable Fn III domains in addition to the eight constant ones, and a C-terminal FBG-like domain (96). The FBG domains, highly conserved among the Tenascin family members (TNC, tenascin R, tenascin W, and tenascin X), can dock with TGF-β small latent complex and activate latent TGF-β (97). In addition to sharing domains that can be unfolded by cell forces with Fn, TNC can bind Fn directly (98). Analogously to Fn, TNC possesses cryptic sites revealed *via* matrix metalloproteinase degradation. These sites run a gamut of signaling effects, ranging from promoting apoptosis to cell spreading (99). An RGD site on TNC allows binding of integrins, specifically the αv family expressed by fibroblasts. TNC is widely expressed during embryonic development, but in adults, it is only upregulated during wound healing or pathological conditions (100). In fetal wound healing, TNC is deposited much earlier compared with adults (1 *versus* 24 h) and has been positively correlated with the wound closure speed since its appearance precedes and promotes the migration of fibroblast (Fig. 1) (101). On the other hand, TNC has been implied in the persistence of organ fibrosis (102) and its secretion and cellular signaling possibly connected in autocrine fashion. These apparent contradictions might be explained by the property of TNC to sequester soluble factors and affect their signaling (99). It has been previously shown that homobox gene Prrx1 is a transcription factor for TNC (103), but only recent work connects TNC expression with Twist1 signaling (104), which is upregulated in fibroproliferative diseases, including IPF, where it inhibits senescence and apoptosis of fibroblasts (105). TNC then acts as a ligand for toll-like receptor 4, whose downstream effects include collagen I secretion and αSMA expression by myofibroblasts (106). As additional evidence of this interplay, TNC KO mice models showed attenuated pulmonary and skin fibrosis (107). Furthermore, a recent study on a murine myocardial infarction model indicates that TNC deletion suppresses inflammatory macrophages while increasing the anti-inflammatory and tissue remodeling activation states. TNC addition *in vitro* upregulated secretion of proinflammatory cytokines, such as IL-6 and TNFα (Table 2) (108, 109). Given its predominance in fetal wound healing as well as its proinflammatory effects on macrophages, the ability of TNC to bind to Fn and liberate latent TGF-β serves as a mediator between the hemostasis and inflammatory phases. TNC mechanisms in wound healing and chronic repair need to be elucidated further in order to devise targeted approaches.

Altogether, the events and mediators that take place during hemostasis continue to contribute to wound healing by informing the recruitment and activation of immune cells during the inflammation phase, which begins hours after injury and may last for days (Fig. 1).

## Inflammation and innate immune system cells

Inflammation has been subdivided into three phases, with the first being defined by cytokine and chemokine expression, tissue-resident immune cell activation, and immune cell recruitment. Among the relevant inflammatory cytokines are ILs, signaling molecules affecting even nonimmune cells (Table 1). In the second phase, proinflammatory signaling begins to subside and is replaced by profibrotic and repair signaling in effector cells such as IL-4- and IL-13-primed macrophages, which release TGF-β and VEGF among other factors. Even though these ILs facilitate tissue repair, long-term activation of such IL-4 and IL-13 pathways might contribute to pathological fibrosis (18, 19). In the final phase, immune cells either retreat from the wound site or undergo apoptosis, enabling the next steps of the wound healing process. This last step is paramount, since timely curtailing of inflammation is necessary to prevent formation of chronic wounds. In the next sections, we will explore the mediators and cell populations critical to inflammation.

The combination of secreted cytokines, growth factors from activated platelets, and a stiffened provisional matrix pave the way for tissue-resident immune cells to be recruited and contribute to the mobilization and action of immune cells. Tissue-resident immune cells (*e.g.*, mast cells, DCs, and macrophages) are innate immune cells that originate from the bone marrow, mature in the peripheral tissue, and remain there for the duration of their lifetime. These cells demonstrate substantial heterogeneity with tissue-specific niches; yet still serve similar purposes across their different contexts: antigen presentation, clearing cellular debris, eliminating infection, responding to signals of tissue damage, tissue repair, and resolving inflammation (17).

### Mast cells

Mast cells are perivascular innate immune sentinel cells that were first discovered in the tumor bed but have been most well characterized in the context of allergies and asthma. More recent work demonstrated critical roles for mast cells in a variety of contexts. For the purposes of wound healing, mast cells represent a key first line of defense in response to tissue injury by responding to several damage-associated molecular patterns (DAMPs) and initiate the cascade of events necessary to adequately address an injury (17). It is worth specifying that inflammatory responses of DAMPs also take place without wound or pathogen, and those are defined as sterile inflammation (110). Important DAMPs during tissue injury include but are not limited to IL-33, heat shock proteins, ATP, and nucleic acids. Mast cells are uniquely equipped to respond to any of the aforementioned DAMPs and other mediators that promote activation and degranulation by releasing preformed mediators stored in their granules, which consist of histamine, arachidonic acid metabolites, proteases, cytokines, and growth factors (Fig. 1) (111, 112, 113, 114, 115). DAMPs alone are not sufficient to induce mast cell degranulation but can stimulate *de novo* synthesis of cytokines, chemokines, and growth factors, including IL-1β, IL-4, IL-6, IL-10, IL-13, TNF, monocyte chemoattractant protein-1, macrophage inflammatory protein-1a, VEGF, TGF-β1, and βFGF (Table 1) (116). The functional effects of mast cells in wound healing can be seen in studies using murine fetal models. Mast cell-deficient fetal mice have fewer scars in a model of dermal wound healing compared with wildtype controls (117). Furthermore, in scarless wound healing, mast cells are fewer, less mature, and degranulate less (118). The effects of some DAMPs in murine models implicate mast cells in the fibrotic process. IL-33 was elevated in scar-forming wounds among fetal mice, and IL-33 injections prompted more collagen synthesis among fibroblasts in both adult and fetal mice (119). Similarly, high mobility group box 1 (HMGB1) is elevated in scar-forming wounds in fetal mice. Fetal mice that would heal scarlessly instead develop scars when treated with HMGB1 (120). Controlled release from mast cells of TNF, IL-1β, IL-6, and other cytokines helps recruit more immune cells to clear out cellular debris and make way for tissue repair. The balance and control of inflammatory *versus* resolving signals not only determines if repair occurs but also informs the behavior of another critical tissue-resident immune cell populations, macrophages.

### Macrophages

Macrophages are critical effector cells of innate immunity that are present in every tissue and serve a protective role in maintaining homeostasis, clearing cellular debris, including apoptotic neutrophils, presenting antigens, and promoting tissue repair. Similar to mast cells, macrophages have DAMP receptors to help make up the first line of cellular defense against infection and injury. Macrophages (and fibroblasts) express DAMP receptor “triggering receptors expressed on myeloid cells-1” (TREM1) (121, 122). Among TREM1 ligands is HMGB1, which can be released by dead cells in sterile conditions (123). While the mechanism has not been fully elucidated, expression of TREM1 is upregulated in fibrosis, which can involve sterile inflammation conditions (123, 124). Macrophages can be tissue resident, originating from early progenitors in the fetal liver and yolk sac, or derive from bone marrow–derived monocytes that infiltrate a wound site. However, recent work has demonstrated a nonvascular route of macrophage recruitment to the site of tissue damage in the liver. Those macrophages originated from the peritoneal cavity and developed a reparative phenotype (125). Macrophages are categorized by tissue location, but more importantly by phenotypic plasticity, since they activate into inflammatory, anti-inflammatory, and tissue remodeling phenotypes that they then can transition between to create a spectrum of macrophages at the wound site (Fig. 3) (17, 126). Recent work shows that agents known to promote inflammatory macrophage activation lead to upregulation of TSP-4. Exogenous delivery of recombinant TSP-4 to macrophages further promoted the proinflammatory phenotype, in an apparent feed-forward loop (88). Macrophages exposed to TSP-4 show both increased accumulation in the site of injury and completion of the transition from releasing of further proinflammatory signals to apoptosis, highlighting the interplay between ECM components and immune cells. Unsurprisingly, embryonic macrophages phenotypically resemble the anti-inflammatory phenotype and are classified as such (127). Since an apparent lack of prolonged inflammation could contribute to scarless wound healing, much recent work leveraged the anti-inflammatory state to promote healing and repair (128, 129, 130, 131).

In addition to ECM–macrophage signaling, interplay between macrophages and fibroblasts in wound healing has become a new focus for the field. IL-4- and IL-13-primed macrophages that also express arginase 1 were reported to compete with activated fibroblasts for local l-arginine, causing less collagen deposition, thus assuming a protective role in liver and lung fibrosis (132, 133, 134, 135). In addition, inhibition of IL-13 causes downregulation of arginase 1 (Table 1) (136). *In vitro*, macrophages use integrin α2β1 and stretch-activated channels to sense mechanical cues associated with tissue remodeling, specifically a deformation of collagen matrices induced by contracting fibroblasts. Mechanical force is sufficient to induce macrophage migration to the site of remodeling, independent of collagen condensation or fiber alignment effects (137). At the site of remodeling, macrophages secrete TGF-β to stimulate activation of myofibroblasts. In both human and murine fibrotic tissues, this activation state is maintained *via* direct contact enabled by macrophages' cadherin-11 (138). In addition to their ability to directly stimulate myofibroblasts, recent work has shown that macrophages are capable of transitioning into smooth muscle cells or myofibroblasts in the context of atherosclerosis, subretinal, or renal fibrosis, respectively (139, 140, 141). In conclusion, macrophages are complex and jack-of-all-trades components of the innate response, but their direct interaction with fibroblasts should serve as motivation to manipulate and further understand their role in scarless wound healing and fibrosis.

### DCs

DCs serve as the key antigen-presenting cell in the peripheral tissue and serve as the bridge between innate and adaptive immunity. They have been shown to be present in wound sites, and the wound healing process is delayed in their absence. More specifically, DCs were shown to be necessary for early cell proliferation, granulation tissue formation, TGF-β1 levels, and CD31+ vessels (142). In the context of myocardial infarctions, DCs are recruited by day 7 following the infarction and shown to be necessary for remodeling (143). DC ablation further resulted in deteriorated left ventricular function, enhanced inflammatory cytokine expression, elevated matrix metalloproteinase 9 (MMP-9), suppressed IL-10, and elevated inflammatory macrophages (143). Interestingly, fetal DCs have a suppressed response to allogeneic antigens, inhibit T cell–derived TNFα production, and regulatory T-cell induction (144), thus contributing to a subdued inflammation in scarless healing (Fig. 3). After tissue-resident immune cells respond to the initial injury, they quickly facilitate the recruitment of neutrophils to the injury site.

### Neutrophils

Neutrophils are short-lived innate immune cells that mature in the bone marrow and are the first immune cells recruited to the site of injury. After maturation, neutrophils reside in the vasculature and monitor for cues of tissue damage and inflammation. Granulocyte-colony stimulating factor, a potent neutrophil chemoattractant, is released by immune and stromal cells during tissue repair to promote neutrophil recruitment (145). When neutrophil receptor CXCR2 binds to CXCL1 or CXCL2 present at the injury site, this prompts neutrophils to roll and arrest at the injury site and cross the endothelium into the damaged tissue site (Table 1) (145, 146). They clean the wound site through microbial sterilization (release of superoxide and hydrogen peroxide) and clearing out debris, primarily through phagocytosis. While neutrophils are among the first cellular responders at an injury site, persistent or chronic presence of neutrophils in peripheral tissue becomes pathological (147). Neutrophils possess DAMP receptors, including TREM1 (122) and can contribute to sterile or pathogen-induced inflammation by releasing inflammatory cytokines and proteases. These proteases can disrupt growth factor efficacy in wound healing (147). Neutrophils release neutrophil extracellular traps (NETs) by releasing uncondensed chromatin bound to cytotoxic proteins. These NETs can bind to and kill bacteria (Fig. 3) (148). Protein arginine deiminase 4 (PAD4) is a key enzyme in NET formation and, according to recent studies, can also citrullinate (modifying an arginine to a citrulline residue) ECM molecules like Fn, impacting its inflammatory bowel disease (IBD). Genetic loss of this enzyme accelerates wound healing, indicating that inhibiting NET formation may improve wound healing (149). Among neonates, there is neonatal NET inhibitory factor within the umbilical cord blood, and it serves as an endogenous inhibitor of NET formation in fetal wound healing. Neonatal NET inhibitory factor–related peptides have been identified that also inhibit NET formation and may be helpful in controlling inflammation during wound healing (150). As inflammation begins to resolve, the injury site enters the third stage of wound healing, tissue repair.

## Repair and fibroblasts

Synthesis of fibrillar ECM proteins, specifically collagen and cellular Fn that differs from plasma Fn in clot formation, occurs on the order of days to weeks depending on the extent of tissue damage. Notably, complete repair takes place reliably in bone only. In other tissues, original cells proliferate, and the tissue recovers its primary functionality only if the gap is small enough during the process called primary repair. Otherwise, secondary repair takes place, involving further ECM secretion and then the formation of granulation tissue in the wound site.

During primary repair in skin, keratinocytes move from the basal lamina, release MMPs, and upregulate α5β1 and αvβ6 integrins to move through the provisional matrix. Upon reaching the wound location, keratinocytes undergo a proliferative burst that is stopped only by contact inhibition once the wound is closed (151). Secondary repair in skin occurs when re-epithelialization is not sufficient (21, 22). First, dermal fibroblasts migrate into the wound and then secrete Fn-EDA. After TGF-β signaling from macrophages (23), they express αSMA and secrete collagen as well (1). Keratinocytes migrate over the newly produced ECM and release VEGF (25). The ensuing angiogenesis and neovascularization that follows is needed to support myofibroblastic presence, leading to the formation of granulation tissue. In this section, we will cover TGF-β signaling and the defining characteristics of activated fibroblasts, including the integrins that mediate mechanotransduction.

### TGF-β signaling in fibroblasts

The TGF-β superfamily of cytokines comprised 40 members, including three TGF-β isoforms and 21 bone morphogenic protein (BMP) (152). The canonical signaling mechanisms for TGF-β and BMP are similar where both classes of cytokine bind their respective type II membrane receptor, which then recruits and phosphorylates the type I membrane receptor (153, 154). TGF-β has a type III receptor lacking kinase activity (155, 156), whereas TGF-β and BMP signaling through type I and II receptors involves phosphorylation of cytoplasmic transcription factors, chiefly the SMAD class of transcription factors. TGF-β leads to recruitment and phosphorylation of SMAD 2 and 3, whereas BMP signals *via* SMAD 1, 5, and 8. Both SMAD complexes translocate into the nucleus after inclusion of SMAD 4 (152, 154).

Of note, BMP-1 has been shown to cleave TSP-1, enhancing its TGF-β–releasing ability, promoting myofibroblast activation. This phenomenon was reported in human corneal scar (157). While BMP-2 is expressed at lower levels in human fetal skin compared with adults and BMP receptors are expressed only in fetal hair follicles (158), application of exogenous BMP-2 to fetal skin wounds recapitulates adult scar formation, which is characterized by hyperplasia and disorganized collagen fiber deposition of fibroblasts (159). Several secreted BMP antagonists (*e.g.*, noggin, chordin, follistatin, cerberus, dan, and gremlin) bind and prevent BMP interactions with their receptors (reviewed in depth elsewhere (152, 160)). Remarkably, gremlin has been described as a potentiator of TGF-β signaling as it activates SMAD2/3 in the absence of TGF-β, then TGF-β upregulates gremlin, and this dynamic has been observed in kidney fibrosis. This signaling has been demonstrated to increase both epithelial-to-mesenchymal transition and fibroblast activation (154, 161). SMAD2/SMAD3 are the transcription factors for TGF-β signaling, whereas cytosolic SMAD7 has been shown to be an antagonist of TGF-β signaling (162).

BMPs are secreted extracellularly in their active form, whereas TGF-β isoforms are embedded in the ECM in an inactive form as part of the latent TGF-β complex. Activation of matrix-bound TGF-β is often mediated by ECM stiffness, creating a positive feedback loop within the microenvironment of TGF-β responsive and contractile myofibroblasts. TGF-β is of paramount importance in diseases of altered mechanics and wound healing, making it a prime candidate to partially explain how second-trimester human fetal skin can heal without scar (163, 164). This property of fetuses is maintained across other mammals on a shorter time scale, given the quicker gestation periods. Indeed, the underlying differences in the mechanisms of signaling of TGF-β among fetal and adult fibroblasts remain poorly understood.

It has been long established that despite the high degree of homology, the three TGF-β isoforms have different effects on scar formation and wound healing. A seminal article by Shah *et al.* (165) showed that using antibodies to inhibit TGF-β1 and TGF-β2 or adding exogenous TGF-β3 reduces scarring in (adult) rat cutaneous wounds. TGF-β1 (and to a smaller extent, TGF-β2) is considered profibrotic and found in hypertrophic scars; moreover, administration of TGF-β1 in early gestational fetal wounds provokes scar formation (166). TGF-β3 is not absent in adult wound healing, but it is highly upregulated after about a week (167), when compared with fetal conditions. In a model of embryonic mouse cleft lip, TGF-β3 transcript was upregulated within 10 min and increasingly expressed 30 min after surgical repair (Figs. 1 and 3; Table 2) (168). TGF-β3 is necessary (169, 170) but not sufficient to explain the scarless wound healing phenomenon in fetuses, since its transcript levels do not change drastically while embryos lose their scarless capabilities (between E16 and E18 in rats) (171, 172).

Indeed, fetal skin contains latent TGF-β complex in matrix (173). Overall, total isoforms of TGF-β expression are lower in unwound fetal skin, and the composition differs from the adult. Specifically, there are slightly higher amounts of TGF-β2 and detectable amounts of TGF-β3, which is hypothesized to have antifibrotic effects and is expressed for prolonged periods in fetal scarless healing (174, 175, 176). On the other hand, the adult TGF-β set is dominated by TGF-β1, which is mostly linked to fibrotic outcomes. During fetal scarless wound healing, the overall ratio of TGF-β3/TGF-β1 is much higher than in adults, with TGF-β3 contributions from keratinocytes and fibroblasts (177, 178, 179, 180, 181). These findings suggest that TGF-β isoforms and their relative amounts play an important role in the scarless wound healing process typical of fetuses, based on the observed findings in multiple studies. Other fibroblast-specific signaling and phenotypes might offer some clues.

LEM domain–containing protein 3 (LEMD3) is an integral nuclear protein biophysically connected to the cell's cytoskeleton, which is constantly remodeled in an ECM stiffness-dependent fashion. LEMD3 antagonizes TGF-β1 signaling by sequestering SMAD2/3 *via* its C-terminal RNA recognition motif and promoting its dephosphorylation and translocation outside the nucleus. It has been shown that fibroblasts' LEMD3–SMAD2/3 interactions were inversely correlated with ECM stiffness and TGF-β-driven transgene activity. In addition, it was discovered that LEMD3 can be proteolytically modified to create cytosolic forms containing the SMAD-binding domain of the full-length protein. These results were confirmed by detection of cytosolic interactions between SMAD and LEMD3 in human tissue. Specifically, lung fibrotic tissue showed locally diminished inhibitory interactions and cytoplasmically shifted LEMD3–SMAD2/3 interactions, in agreement with *in vitro* assays (182) (Fig. 4). Even though this work focused on adult fibroblasts (182), lower amounts of LEMD3 being cleaved and released in the cytoplasm could explain the different sensitivity of fetal fibroblasts to TGF-β. If that were the case, LEMD3 bound to the nuclear envelope would be affected by cytoskeletal strain because of increased substrate stiffness (typical of *in vitro* conditions) and limited in its antagonizing capability of SMADs.

Lower physiological ECM stiffness and altered LEMD3 antagonism of TGF-β would not be the first instance of different pathways in fetal fibroblasts. A study on fetal- and adult rabbit–resolving wounds revealed that fetal fibroblasts accumulate collagen I by proliferation without upregulating collagen mRNA, whereas adult wounds displayed fibroblastic migration and then significant upregulation of collagen I transcription. These findings suggest that fetal fibroblasts are capable of proliferating and secreting collagen at the same time, unlike their adult counterparts, potentially contributing to a shorter healing time (183).

In fetal fibroblasts, the typical autocrine loop of TGF-β1 autoinduction is absent (184), and collagen-binding integrins (α1 and α2) appear to be downregulated in response to TGF-β isoforms (185). Moreover, TGF-β1 protein is rapidly overexpressed in fetal mouse embryo injury models but is cleared out by 18 h (186). This limited window reduces TGF-β1 activation of scar-forming pathways, indicating reduced fibrosis. Given the evidence of different pathways in fetal fibroblasts reported here, the levels of interaction between cytoplasmic-cleaved LEMD3 fragment and SMADs should be investigated. Lack of the cytoplasmic fragment would reinforce the dependence of LEMD3 action on substrate (and ECM *in vivo*) stiffness, which is known to be lower in fetuses. Altogether, these data indicate new targets and avenues of investigation in TGF-β signaling.

### Wingless type

Another key signaling pathway in tissue remodeling is wingless type (Wnt), a key regulator in not only embryonic development but also wound healing. The canonical pathway is comprised of Wnt isoforms binding to Frizzled membrane receptor in order to enable beta-catenin accumulation in the cytosol and its translocation into the nucleus as a transcription factor. In the absence of the ligand-binding event, glycogen synthase kinase-3β phosphorylates the N terminus of beta catenin, leading to its ubiquination for degradation. This pathway is also affected by TGF-β signaling mediators (187), such as the Wnt antagonist Dkk-1, which is downregulated by TGF-β expression (188) (Fig. 4).

Studies suggest that excess Wnt signaling is responsible for endothelial to mesenchymal transition in keloids and dermal scars (189, 190, 191). Moreover, accumulation of beta-catenin was detected in fibroblastic foci of IPF lung (192, 193). These findings indicated Wnt as another prime target for fibrotic diseases, spurring several attempts to alter the pathway to achieve scarless healing (194). Recent work attempted to reduce scar formation of animal dermal wound models *via* topical application of a variety of Wnt and catenin inhibitors (195). *In vitro* transfection of CXXC5 (a zinc finger catenin inhibitor) reduced αSMA and collagen I expression in fibroblasts (191), whereas in mice models, topical applications of XAV-939 and pyrvinium also led to the formation of structures typical of intact skin and a reduction in fibrosis (196).

Further investigation of Wnt signaling is necessary because similar to TGF-β, different isoforms of Wnt have almost opposite downstream effects. Dosing of Wnt3a increased proliferation and collagen I production in postnatal murine fibroblasts but not in fetal. Furthermore, the same Wnt isoform increases TGF-β1, whereas decreasing TGF-β3 (antifibrotic) secretion in fetal fibroblasts and even more so in postnatal ones (197). On the other hand, Wnt6 expression reduced epithelial-to-mesenchymal transition in response to TGF-β dosing, indicating a broader protective effect (Table 2) (198). Wnt signaling provides different therapeutic targets that could help suppress fibrosis.

### Characteristics of fetal and adult myofibroblasts

Myofibroblasts, activated fibroblasts that assemble, contract, and stiffen ECM, are thought to be the culprit of fibrotic disease progression. Unfortunately, myofibroblast remodeling of the microenvironment drives further differentiation of naive fibroblasts as a consequence of their adaptation to the stiff microenvironments generated by myofibroblasts. In fibrotic conditions, these cells show an increased resistance to apoptosis, fueling this positive feedback loop of ECM secretion and force generation *via* αSMA expression (24). Given their importance and the current insufficient definition based on *in situ* morphology (199), it would be ideal to first characterize and then precisely target myofibroblasts in order to address fibrotic progression that can be considered a consequence of dysregulated wound healing.

Unlike the adult counterparts, fetal fibroblasts demonstrate low levels of background αSMA expression (185). Under physiological conditions, fetal wounds (and skin) are more compliant (equivalently, they are softer) than adults', which might be due to lower ECM crosslinking levels and more collagen III (200, 201, 202). ECM secreted by fetal (myo)fibroblasts contains more collagen III and is organized in a weave pattern, similar to uninjured ECM. As such, fetal wounds regain all their mechanical strength unlike adult or postnatal scars. For comparison, adult fibroblasts secrete collagen III only in the beginning stages of repair. Later, collagen I becomes the dominant form and makes up most of the collagen in the wound ECM (203). As a suggestion, myofibroblasts present in fibroblastic foci in IPF and involved in ECM remodeling express prolyl 4-hydroxylase for collagen crosslinking, which could serve as another marker of myofibroblasts in fibrosis (204). The differential composition of fetal ECM might further impact phenotype of fibroblasts because collagen I and collagen III preferentially ligate different integrins leading to divergent downstream signaling.

Fetal and adult wounds also differ on the basis of hyaluronan expression and signaling. This glycosaminoglycan was historically regarded as one of the main factors enabling a scarless healing in embryos below a certain gestational age. The work of Longaker *et al.* (205) established the lengthier presence of hyaluronic acid (HA) in the fetal wound, up to 3 weeks, compared with adult wounds where it disappeared within 1 week. A subsequent study highlighted the increase in HA receptors, namely CD44 and RHAMM, in both fetal and adult fibroblasts. However, a mismatch between too few receptors on adult fibroblasts and the abundance of the ligand, HA, might contribute to scar formation, since the upregulation of CD44 is more elevated in response to wounds in fetal fibroblasts (206). More recently, the mechanism connecting IL-10 with HA synthesis *via* STA3 signaling was discovered and tested *in vivo*, which suggests a new role for IL-10 beyond its accepted immune-regulatory mechanism. Fibroblasts exposed to IL-10 shift to an antifibrotic phenotype, thanks to the formation of a HA-rich wound ECM (207). Furthermore, IL-4 and IL-13 signaling appears to induce expression of CD44 in fibroblasts (208), reinforcing the role of HA in wound resolution (Table 2). Recent *in vitro* work has shown that soft HA gels with an integrin ligand such as collagen I stimulate PI3K/Akt signaling and formation of stress fibers in immortalized fibroblasts to a degree comparable to much stiffer polyacrylamide gels (209). This could possibly indicate an activated (myo)fibroblast phenotype playing a role also in scarless, *in vivo*, wound healing. Despite the simplistic view that soft microenvironments are necessary to reduce scar formation, as in fetal wound healing, the increased signaling and cytoskeletal remodeling on HA gels indicate the importance of the modulation of contractile machinery of fibroblasts in wound repair, as discussed earlier in this section. Further investigation of this phenomenon *via* studies that take HA viscoelastic substrates into account is also warranted (210).

### Thy-1

Myofibroblasts contributing to fibrosis might be better defined by a surface marker they do not express, to distinguish them from fibroblasts in homeostasis. Thy-1/CD90 is another important factor in the mechanotransduction of myofibroblasts. It was first discovered as a thymocyte marker (hence its name, thymocyte differentiation antigen 1) and used as an antigen to identify leukemia cells (211). Thy-1 expression was later observed in T cells, endothelial cells, fibroblasts, smooth muscle cells, and mesenchymal stem cells. Thy-1 is a GPI-anchored protein that also contains an RLD domain (which mimics the RGD integrin-binding sequence) and organizes lipid rafts and engagement of αvβ3 and αvβ5 (212, 213). Our group has recently shown that Thy-1 loss in fibroblasts promotes dysfunctional mechanotransduction and rigidity sensing (212). While exposure to soft substrates normally inhibits myofibroblast differentiation, Thy-1–deficient fibroblasts on soft substrates overcame that inhibition by disrupting mechanosensitive force-induced signaling and promoting myofibroblastic differentiation. This led to enhanced ECM protein deposition and remodeling that subsequently lead to cell stiffening (212). Furthermore, previous work has shown that Thy-1 (−) and α-SMA (+) fibroblast subpopulations are localized to fibrotic lesions in IPF (204). The role of Thy-1, in fibroblast biology in particular, gathered much interest after it was noted that stable subpopulations of resident fibroblasts were present in the murine lung and differed on the basis of surface Thy-1 expression. Early observations noted that Thy-1 negative (−) lung fibroblasts demonstrated a more spread morphology with reorganized cytoskeletal proteins and stress fiber formation (214) plus were susceptible to contraction-induced latent TGF-β1 complex activation and myofibroblast differentiation. Thy-1 specifically interacts with integrin αvβ5 to prevent its engagement of the latent TGF-β1 complex (Table 2) (213).

Some work has been done attempting to determine the mechanism of Thy-1 loss in fibroblasts. Lung tissue sections from patients with IPF show that fibroblasts within fibrotic lesions have a hypermethylated Thy-1 promoter; yet DNA methyltransferase activity did not seem to explain how the Thy-1 promoter was hypermethylated (215). The contribution of epigenetics was explored further when it was shown that using a histone deacetylase inhibitor, trichostatin A, restored Thy-1 expression and reversed the hypermethylation of the Thy-1 promoter (216). When used *in vivo*, the histone deacetylase inhibitor suberoylanilide hydroxamic acid dampened bleomycin-induced fibrosis in mice (217). Another possible mechanism for Thy-1 loss that has been implicated is vesicular shedding. It has been shown that among lung fibroblasts that have lost Thy-1 expression *in vitro*, the media supernatant contained Thy-1 with an intact GPI anchor. The intact GPI anchor seems to convey that Thy-1 is still membrane bound, potentially pointing to a role for Thy-1 being shed onto extracellular vesicles (218). Thy-1 presence has been detected *via* flow cytometry in fetal fibroblasts (219), but no quantization was provided in that study (Table 2). Given our current understanding, we predict that fetal fibroblasts possess Thy-1 throughout the wound healing process, potentially another factor contributing to scarless resolution.

### Proteins similar to Thy-1

As we highlighted previously, Thy-1 is an important regulator of fibroblast mechanotransduction, but little is known about the role of similar proteins with a GPI anchor and RLD analogous site, such as SEMA7A (semaphorin 7A) and CNTFR (ciliary neurotrophic factor receptor). The similar structure of such membrane proteins suggests that cells have alternative mechanisms to compensate for Thy-1 loss or perhaps interact with integrin to alter mechanosignaling in an entirely new way (220).

Given the continued search for a defined myofibroblastic marker and the debate in the field, the best approach to identify myofibroblasts should rely on a combinatorial approach of phenotypical functions, which are not limited to the molecules listed previously.

### Integrins of (myo)fibroblasts

In addition to biochemical stimuli, fibroblasts respond to mechanical cues from their environment, in a process defined as mechanotransduction, which has been implicated as a key axis driving activation of naive fibroblasts down a myofibroblastic lineage. Mechanotransduction is a cell phenomenon involving conversion of physical cue signals into biochemical signaling. This can occur at the level of individual proteins (molecular) that undergo conformational changes in response to force and at the level of cellular/subcellular macromolecular complexes, which both transmit and translate forces through activation of key signaling pathways (221). The primary force-sensing apparatus at the fibroblast–ECM interface is the focal adhesion (222), comprised of integrins specific to particular motifs within various ECM proteins, on the extracellular side. On the cytoplasmic side, focal adhesions also consist of a plethora of adaptor proteins and key kinases, like focal adhesion kinase (FAK) and Src family kinase (SFK) among others (223). Importantly, there is recent evidence that specific integrins, such as αvβ3, may be potentially pathological mechanotransducers, whereas others, such as α5β1, may play more fundamental structural roles during normal physiology (224). In the following sections, we will discuss the integrins expressed by (myo)fibroblasts, grouped on the basis of their binding site and ligand (Table 3).

**Table 3:** Integrins of myofibroblasts

Integrin	Ligand and binding site	Function	Involved in
α1β1	GFOGER, but GLOGEN on collagen III, is more prominent (245, 246, 247)	Binding downregulates collagen secretion (248)	Fetal scarless healing
α2β1	GFOGER on fibrous collagen (245, 246)	Binding upregulates MMP1 and MMP13, could promote NF-κB (248, 251, 252, 253)	Scar formation
α4β1	Fn extra fomain A (242, 316), TSP-1 (241)	Overlaps with α9β1, plus migration and proliferation (243)	Granulation tissue (scar)
α5β1	RGD plus PSHRN synergy site on Fn (54)	Cell homeostasis and reinforces adhesion (45, 231)	Resolved wound healing
α9β1	Fn extra domain A (242, 316), TSP-1 (236), TNC (240)	Filopodia formation, Cdc42 signaling (237, 238, 239); promotes angiogenesis *via* TSP-1 binding (236)	Granulation tissue (scar)
α11β1	GFOGER on fibrous collagen (245, 246)	Overexpression leads to phenotype resembling cardiac fibrosis (260), can liberate TGF-β from LAP *via* TNX (257)	Granulation tissue (scar)
αvβ1	RGD on Fn, LAP complex of TGF-β (226, 227)	Can liberate TGF-β from LAP (226, 227); its inhibition prevents fibrosis (230)	Fibrosis
αvβ3	RGD on Fn, LAP complex of TGF-β (56, 226, 227)	Outcompetes α5β1 for Fn binding. Interacts directly with Src (231, 232)	Fibrosis, cancer
αvβ5	RGD on Fn, LAP complex of TGF-β (226, 227)	Can liberate TGF-β from LAP (226, 227)	Fibrosis
αvβ6	RGD on Fn, LAP complex of TGF-β (226, 227)	Canonically liberates TGF-β from LAP (173, 226, 227)	Fibrosis
αvβ8	RGD on Fn, LAP complex of TGF-β (226, 227)	Can liberate TGF-β from LAP (226, 227)	Fibrosis

Abbreviations: GFOGER, glycine–phenylalanine–hydroxyproline–glycine–glutamate–arginine; LAP, latency-associated peptide.

### αvβ6, αvβ1, and TGF-β

As discussed earlier, TGF-β isoforms have a multifaceted impact on scarring and granulation tissue. TGF-β is deposited in the ECM in the latent TGF-β complex. As such, its activation by cells is intrinsically affected by the matrix stiffness. To release the cytokine from its latent complex, cells mechanically free TGF-β *via* stresses mediated by αvβ6 integrin, enabling TGF-β paracrine signaling. Stiffer ECM augments the forces transmitted to the latent complex, increasing the liberation of TGF-β. This enables a feed-forward loop, in which TGF-β signaling stimulates ECM protein secretion and cellular stresses, furthering the stiffening of the matrix (Fig. 4) (173, 225).

In addition to αvβ6, other fibroblast αv integrins (αvβ1, αvβ3, αvβ5, and αvβ8) can bind to the RGD sequence of the latent TGF-β complex and liberate all TGF-β isoforms (Table 3) (226, 227), except for TGF-β2. The latter isoform lacks the RGD motif, likely relying on mechanically deformation to release TGF-β2 (228, 229). Reed *et al.* (230) discovered a specific and potent inhibitor of αvβ1 and used it to show protective effects against lung and liver fibrosis in *in vivo* mice models. Moreover, unless αvβ1 was specifically inhibited, several cell lines including lung fetal fibroblasts managed to access the latent TGF-β complex and liberate TGF-β despite administering an antibody blocking other αv integrins. This suggests that αvβ1 could also be the dominant integrin in scar formation to release ECM-bound TGF-β.

### αvβ3 and α5β1

These two integrins bind the canonical IBD of Fn, defined by the accessible RGD peptide sequence on the 10III repeat. While this ligand is sufficient for αvβ3 binding, α5β1 also requires the PSHRN peptide sequence on the 9III repeat, the “synergy site,” in close proximity (Table 3). Recent studies (231) indicate that the αvβ3 integrin in physiological conditions outcompetes α5β1 when initiating binding on Fn but afterward recruits α5β1 to strengthen adhesion. Despite advances in understanding mechanotransduction, experiments to better define the effects of the integrin switch still have to be performed, particularly in the context of Fn conformation and fibroblasts in fibrosis. Src, a member of the SFK, binds constitutively to β3 (232) and is activated following integrin engagement (Fig. 2). This allows Src to bind to and promote the activity of one of its downstream targets, FAK. FAK can phosphorylate paxillin, a molecular scaffold for other focal adhesion proteins, that when interacting with adapter molecule Crk (p38) leads to Rac1 activation and downstream F-actin polymerization (223). Moreover, active Src can promote phosphorylation of kindlin-2 and form a complex with it, which in turn promotes phosphorylation of paxillin (233). What is critical to myofibroblast differentiation is that these events lead to enhanced polymerization of F-actin, which in turn drives the translocation of myocardin-related transcription factor (normally cytoplasmically sequestered by G-actin) into the nucleus, where after complexing with serum response factor, instructs transcription of myofibroblastic and fibrosis-relevant genes. These pathological programs can be offset by conditionally knocking out or inhibiting integrin αv, resulting in protection from various solid organ fibrosis (234). Interestingly, recent work on cancer ECM shows that fibroblasts with inhibited, knocked out, or dominant negative FAK regained myofibroblastic development when an α5β1-inhibiting antibody was administered (235). Src, Fyn, and Yes (all SFK members) null fibroblasts instead did not show myofibroblastic traits after α5β1 inhibition, suggesting nonequivalent function for Src and FAK (235).

### α4β1 and α9β1

The integrins described here bind not only Fn in domains other than the IBD but also TSP-1. Engagement of α9β1 with TSP-1 promotes angiogenesis (236), and this signaling might contribute to the timely resolution of inflammation described earlier that requires TSP-1 (84). Both integrins α4β1 and α9β1 bind Fn-EDA (Fig. 2). As mentioned earlier, this domain is present only in the Fn secreted by fibroblasts *in situ*. Engagement of EDA with α9β1 leads to formation of filopodia, which fibroblasts use to probe substrate stiffness (237) and activate Cdc42, a GTPase involved not only in migration and cytoskeletal remodeling but also in the cell cycle progression. In addition, blocking α9β1 *via* a specific antibody slows and reduces formation of granulation tissue during skin wound healing, as shown in excisional wound mice models without altering wound closure time (Table 3) (238, 239). This phenomenon is arguably closer to the ideal scarless wound healing because more granulation tissue leads to less tissue functionality being restored. In addition, α9β1 can also bind TNC on the AEIDGIEL motif (240, 241). This integrin with multiple ligands seems an attractive target for therapeutic approaches, whether by promoting its binding to TSP-1, TNC, or to prevent Fn-EDA signaling. Nakayama reports that α9β1 blocking does not reduce fibroblast migration and proliferation, whereas the lack of effect might be explained by an α4β1 compensation mechanism. Indeed, as α4β1 shares 39% of its amino acid sequence with α9β1 (242), it is not surprising that they bind the same site on Fn-EDA. Given the effects of the EDA domain (including increased stress fiber formation and phosphorylation of myosin light chain kinase, which furthers Fn synthesis and fibrillogenesis), blocking α4β1 with a specific antibody or knocking its expression down *via* siRNA indicates a significant reduction of this profibrotic progression, thus suggesting α4β1 as a viable target for a future antifibrotic therapy (243).

### α1β1, α2β1, and α11β1

All these integrins bind, among other ECM proteins, fibrillar collagens, including type I and III, which are the most abundant collagens secreted during formation of the provisional matrix and fibrosis (Table 3). It has been hypothesized that these integrins have limited binding sites under physiological conditions because of the tight alpha helices that make up collagen (244). The binding sites would become accessible after injury, during development, or during the secretion and assembly of the provisional matrix. While it has been shown that all three integrins bind the collagen consensus GFOGER (glycine–phenylalanine–hydroxyproline–glycine–glutamate–arginine) sequence (245, 246), more recent *in vitro* studies indicated GLOGEN as the more potent binding site of α1β1 (247). Since the latter peptide sequence is present on collagen III and α1β1 engagement downregulates collagen secretion (248), it can be hypothesized that such binding events play a role as a negative feedback loop during fetal scarless wound healing, where collagen III is more abundant than in adult (249, 250). Integrin α1β1 downregulates expression of MMP13 *via* a Raf-1, mitogen-activated protein kinase/extracellular signal–regulated kinase 1/2, extracellular signal–regulated kinase 1/2 pathway (251). On the other hand, α2β1 engagement on collagen upregulates MMP1 (248) and MMP13 (251). Increased expression of the latter is due to mitogen-activated protein kinase kinase kinase, mitogen-activated protein kinase kinase 3/6-mediated activation of p38. Enhanced production of MMPs is typically correlated with increased migration and invasion of fibroblasts at first, then increased provisional matrix remodeling. It has been suggested that α2β1 engagement in 3D collagen I lattices also promotes NF-κB signaling, possibly *via* PKC-zeta (252) or PI3K, thus upregulating the same collagen I (253). For the aforementioned reasons, activation of the downstream α2β1 effectors could be considered as an undesired progression of wound healing toward fibrotic characteristics. However, the effects of this integrin on fibrosis might be tissue specific. While KO mice models of glomerular injury displayed reduced granulation tissue *via* negative regulation of collagen synthesis (254), *ex vivo* assays showed a significantly reduced α2β1 expression in IPF fibroblasts when compared with control. In the latter case, higher levels of inactive glycogen synthase kinase-3β were found because of reduced protein phosphatase 2A activation (255).

α11β1 is an integrin present only in a subset of all fibroblasts, yet it is the major collagen I receptor in dermal fibroblasts and is involved in TGF-β signaling. This integrin is largely induced in excisional wounds, but KO models indicate reduced formation of granulation tissue (256). Interaction of α11β1 with the FBG domain of tenascin X (another member of the Tenascin family) was demonstrated to be a prerequisite for TGF-β1 activation from the latent complex (257). TGF-β1 been shown to regulate integrin α11 *via* not only its downstream partners SMAD2/3 (258) but also by noncanonical Jun N-terminal kinase–dependent TGF-β signaling, which was shown to be crucial for α11β1-dependent collagen secretion. Although more information is necessary to link Jun N-terminal kinase activation with α11β1, Gullberg *et al.* (259) suggested TGF-β–activated kinase 1 as a potential candidate. More recently, overexpression of α11 in a transgenic mouse model led to a phenotype closely matching cardiac fibrosis (260). These findings implicate integrin α11β1 as a potential therapeutic target by blocking it in the context of myofibroblast differentiation during fibrosis (239).

Further studies of these collagen-binding integrins relying on integrin-KO mice might be hindered by the β1 subunit, which is shared by several other integrins. Nonetheless, in the light of differential fetal fibroblast integrin expression, elucidating which integrin signaling contributes the most to the healing process would provide the field with a druggable target for the pursuit of scarless wound healing in adults. Following TGF-β signaling, myofibroblast activation, and integrin engagement with the ECM during repair, we turn to the final phase of wound healing, remodeling.

## Remodeling

The last phase in a complete wound healing process is remodeling, which lasts from weeks to months. Remodeling takes place except under particular conditions leading to scarless healing such as small dermal wound or in the fetus. Fetal healing conditions do not require realignment of collagen III-rich ECM because it is already deposited in a pattern similar to the original functional ECM. Remodeling canonically begins once fibroblasts are activated into myofibroblasts and begin contraction (24). Myofibroblasts increase expression of actin stress fibers and integrins, in order to produce the contraction needed to realign the excessively deposited ECM (Fig. 1). Activated fibroblasts are thought to be regulated by the Rho-ROCK pathway (261) and continue to support the mechanical load until the ECM is crosslinked, creating striated scar tissue, which in skin becomes paler as time goes by and vascularization is lost. Dermal features such as hair follicles and sweat glands that were lost because of injury are not replaced (6). It is reported that maximum mechanical strength of the scar will be 80% of that of the original tissue, at best. Repaired dermal wounds, especially in people of African or Asian heritage, may progress beyond a typical scar, giving rise to keloid formation (262, 263).

Because of the myriad of factors and cell types involved, a sustained imbalance of either can lead to fibrosis, in which fibroblasts proliferate *in situ*, are activated into myofibroblasts, and continuously secrete ECM. In the following sections of this review, we will highlight cytokines, ECM proteins, and other factors that deviate from conclusive wound healing toward fibrosis (chronic scarring) and the potential opportunities arising from fetal fibroblast research.

### Cytokines and granulation tissue

Granulation tissue is characterized by re-epithelialization, vascularization, and tissue maturation *via* new ECM deposition and fibroblast proliferation. Furthermore, fibroblasts differentiate into myofibroblasts, which mediate wound contraction and continue secreting ECM proteins during remodeling. Each of these processes are facilitated by the introduction of cytokines, chemokines, and growth factors (264). Subcutaneous delivery of TGF-β1 has been shown to promote the development of granulation tissue that is abundant with αSMA+ myofibroblasts. TNFα, IL-1β, and PDGF are also present in granulation tissue and contribute to (myo)fibroblast proliferation (265). Usually, tissue damage results in local hypoxia because of damage to the underlying vasculature. Acute hypoxia contributes to conclusive wound healing since low oxygen tension levels promote angiogenesis, epithelialization, *de novo* ECM production, and fibroproliferation (Fig. 1). Key among the cytokines produced in response to hypoxia is VEGF, which possesses a hypoxia-response element in its promoter and is a potent angiogenic cytokine. In mice lacking a full VEGF promoter and thus deficient VEGF expression, PDGF-BB and stromal cell-derived factor-1α expression were lower, and this corresponded to poor angiogenesis and granulation tissue formation (266). Inhibition of IL-1 signaling *via* IL-1 receptor antagonist (IL-1Rα) decreases secretion of inflammatory cytokines (*e.g.*, IL-1α, IL-1β, IL-12, interferon γ), myofibroblast numbers, and proliferating cells (Table 1). In addition, IL-1Rα expression corresponded to elevated IL-10 expression, larger blood vessel lumen size, increased anti-inflammatory macrophages' activation, and larger granulation tissue size (267). Granulation tissue fibroblasts have elevated expression levels of α5 integrins (bind to Fn), yet lower levels of α1 integrins (binds to collagen and laminin). This finding potentially indicates a preference among granulation tissue fibroblasts for an Fn-rich matrix independently of cytokine treatment or overexpression (PDGF, IL-1β, TNFα, or interferon γ) (268). Collagens, Fn, and other ECM proteins are not only simply passive structural elements but also can contribute to overall homeostasis through the signaling pathways they activate. However, modifications to the ECM occur during remodeling that can alter which downstream pathways are activated as well as subsequent wound healing outcomes.

### Oxidative stress and post-translational modifications

*In utero*, the amniotic fluid and subdued inflammation (because of immune privilege) are able to minimize oxidative stress. However, oxidative stress plays a significant role in adult wound healing culminating in fibrosis. Further research on redox-mediated protein modifications mentioned in this section might enable the reduction of fibrosis without having to recapitulate the amniotic environment. For an exhaustive review on free radicals in the fibrotic context, we recommend the piece by Grosche *et al.* (269).

Oxidative stress and associated free radical reactions cause post-translational modifications in both cytoplasmic and ECM proteins, altering traditional signaling networks. For example, nitrosylation of caspase-3 prevents apoptosis of human peritoneum myofibroblasts in fibrosis (270). Large structural proteins including titin (271) and Fn (272) with the two unpaired cysteines in the 7III and 15III (Fig. 2) can form a reversible bond between glutathione and their unpaired cysteines (amino acids with one thiol group), if these residues are exposed. This modification leads to mechanical changes that may alter downstream fibroblast signaling. Moreover, Fn can undergo another post-translational modification, citrullination of its arginine residues, causing an enhancement in both focal adhesion turnover and fibroblast migration (273). The characterization of PAD2 and PAD4, the citrullination enzymes mentioned in the “Neutrophils” section, led to discovering inhibitors that are now available on the market. Administering such drugs to patients could reduce Fn citrullination and curb fibroblast migration into the wound area.

TGF-β1 signaling is also affected by redox stress. Specifically, the latent TGF-β complex can release TGF-β1 without mechanical stimulation, thanks to an hydroxyl-induced modification of methionine 253 (274). This redox switch likely initiates or compounds the effects of TGF-β1 signaling in fibrotic diseases characterized by redox stress. Overall, further investigation into ECM modifications and downstream outcomes during fibrosis is needed in order to identify potential therapeutic avenues.

## Perspectives and conclusions

We would like to conclude this work with concepts and molecules that were not covered in the main sections. These recent findings and a little leap of imagination should serve as launching pads to spark novel investigations into the dynamic relationship between fibroblasts, the ECM, and inflammation during wound healing.

### Fibroblast heterogeneity

While the field has focused on αSMA and Fn-EDA expression to differentiate myofibroblasts from fibroblasts, recent work shows that these markers might not be sufficient to assure complete targeting of ECM remodeling cells in pathological conditions, because some myofibroblastic subpopulations might not express these markers, and the markers are not exclusive to fibroblast (275). Thus, the field has moved to demonstrate the presence of critical fibroblast subpopulations with nonoverlapping functions, origins, or locations. While the degree of heterogeneity may differ based on tissue origin, fibroblasts across different organ systems appear to possess subpopulations that play a unique role in contributing to scar formation and pathological tissue repair.

Croft *et al.* (276) used a serum transfer–induced model of arthritis to characterize fibroblast heterogeneity. Here, they identified five different fibroblast subpopulations and determined the fibroblast activation protein-α+ Thy-1+ fibroblasts to be critical in disease progression. Identifying which specific subpopulation to target in fibrosis, however, will still require further investigation as fibroblast activation protein-α+ Thy-1+ fibroblasts comprised four of the five subpopulations discovered in this study. Fibroblast heterogeneity has also been investigated in the context of adipose regeneration, an aspect of dermal scarless wound healing, using a full-thickness dermal wound healing model. Findings from this study indicated the presence of wound myofibroblasts with hematopoietic and myeloid origins. Furthermore, the myeloid-derived myofibroblasts are able to revert to *de novo* adipocytes. It should be considered that these myeloid-derived myofibroblasts could have been fibrocytes or macrophages undergoing macrophage-to-myofibroblast transition but are certainly a cell population that has promise as a cell-based therapy and deserves of further study (277).

Lung fibroblast heterogeneity has garnered a great deal of attention recently, especially with respect to pulmonary fibrosis. Using the bleomycin-induced lung fibrosis model, Xie *et al.* (278) demonstrated that lung mesenchymal cell heterogeneity shifts in response to bleomycin. Notably, a PDGFRβ Hi subpopulation that also expresses αSMA emerges and two matrix-producing subpopulations (Col13a1 and Col14a1 matrix fibroblasts) expand. Though it is unsurprising that matrix-producing subpopulations would expand in pulmonary fibrosis, whether both ECM-secreting populations contribute to the pathogenesis of lung fibrosis is worth further investigation in order to potentially target them *via* a novel therapy.

On the other hand, Sun *et al.* (279) showed that cells expressing PDGFRα comprise 95% of the myofibroblasts contributing to fibrotic remodeling in the bleomycin murine model of lung fibrosis.

Others have identified eight subpopulations of fibroblasts in the same model. Out of those, PLIN2+ fibroblast subpopulation may avoid scar formation (280), since it was present among intact alveoli.

While those fibroblast numbers did not change in response to bleomycin, their gene expression profile correlated with bleomycin treatment more than any other cell population (281). When looking at human lung fibroblasts from healthy patients compared with patients with IPF, αSMA+ myofibroblasts were enriched in samples from IPF patients, as expected, but noteworthy was the emergence of an HA synthase 1 Hi fibroblast subpopulation that was only present in IPF samples. The latter upregulated pathway is implicated in epithelial-to-mesenchymal transition and was colocalized with COL1A1 in the subpleural space. All these data point to a potential pathological subpopulation present in human disease that could be helpful to eliminate when trying to promote scarless wound healing.

### Hepatocyte growth factor

While resolving wound healing in adults leads to scar formation outside few circumstances (shallow dermal wound, bone tissue), oral mucosa fibroblasts have shown enhanced capabilities to achieve scarless healing. Recent work by Midgley *et al.* (282) demonstrated that elevated levels of hepatocyte growth factor (HGF) might be responsible for this promising phenotype. HGF has been shown to interfere with TGF-β1 signaling *via* a plethora of mediators, such as SnoN and transforming growth interacting factor (Table 2) (283). However, in depth, studies focusing on the overexpression of any of the three HGF isoforms outside the mucosa environment should be pursued to evaluate its potential therapeutic effect.

### African spiny mouse

Remarkably, the African spiny mouse uniquely exhibits full scarless skin regeneration, making its keratinocytes and fibroblasts prime candidates for the study of scarless wound healing in adult mammals. When compared with the fibroblasts of *Mus musculus*, spiny mouse fibroblasts *in vitro* did not upregulate αSMA, regardless of seeding on soft or stiff substrate, and produced lower traction forces on soft gels. Interestingly, the collagen gels remodeled by spiny mouse fibroblasts in this study are roughly half as stiff as the ones with *Mus musculus* fibroblasts, suggesting lower ECM secretion and crosslinking in the spiny mouse (284). Further studies on the mechanisms enabling this rodent's recovery would be most welcome.

### Conclusions

In this review, we have compared wound healing in adult tissues and fetal conditions in an effort contrast fibrotic *versus* scarless wound healing. Our motivation went beyond the functionality and esthetic concerns with dermal wounds, but rather the inability to restore tissue function after fibrotic remodeling is primarily why so many diseases are tied to fibrosis and why fibrotic complications have been implicated in 40% of the world deaths (285).

The field has not found a single pathway or molecule that could recapitulate scarless healing. This search has been unsuccessful not for lack of trying but may be explained by the dynamic and complex biology present in the wound bed that changes across time, space, cell identity, and mediators. New techniques are enabling us to map epigenetic modifications, single-cell transcript profiles, protein expression levels (single-cell multiomics), and correlation of transcripts with proteins while also retaining tissue morphology information (spatial omics). These multiomic approaches, reviewed at length elsewhere (286, 287, 288, 289), will permit the field to identify and resolve many of these variables. We believe that because of these technological advances, this is the most appropriate time to provide a holistic review of factors tilting the balance toward scarless healing or fibrosis.

While most publications focus on only one of the aforementioned aspects, the sheer amount of significant data from multiomic techniques will facilitate the emergence of patterns and potentially new meaningful markers.

The molecules identified next could favor strategies based on antibody–drug conjugates or borrowing other ligands and conjugate them with a payload, as therapeutic approaches. Potentially more successful approaches might be derived from combinatorial strategies that affect different pathways at the same time. In particular, the field has been making significant progress in achieving reversal of liver fibrosis compared with other solid organ fibrosis (290) Finally, we invite researchers in the field to branch out in order to capitalize on discoveries from other fields in biology or medicine, such as cancer (291) or beyond, that investigate the interplay of fibroblasts, the ECM, and inflammation that might point to novel avenues for discovering new therapeutic targets.

## Conflict of interest

The authors declare that they have no conflicts of interest with the contents of this article.
